# Impedance-Assisted Multivariate Analysis Technique for Enhanced Gas Sensing with 2D Dichalcogenides

**DOI:** 10.1021/acssensors.4c03325

**Published:** 2025-03-31

**Authors:** Bharath Somalapura Prakasha, Peng Xiao, María José Esplandiu, JiaQi Yang, Daniel Navarro-Urrios, Javier Rodríguez-Viejo, Marianna Sledzinska

**Affiliations:** † Catalan Institute of Nanoscience and Nanotechnology (ICN2), CSIC and BIST, Campus UAB, Bellaterra, Barcelona 08193, Spain; ‡ MIND-IN2UB, Departament d’Enginyeria Electrónica i Biomédica, Facultat de Física, Universitat de Barcelona, Martí i Franquès 1, Barcelona 08028, Spain; § Departament de Fisica, Facultat de Ciencies, Universitat Autonoma de Barcelona, Bellaterra, Barcelona 08193, Spain

**Keywords:** TMDs, humidity sensing, impedance, multivariate analysis, neural networks

## Abstract

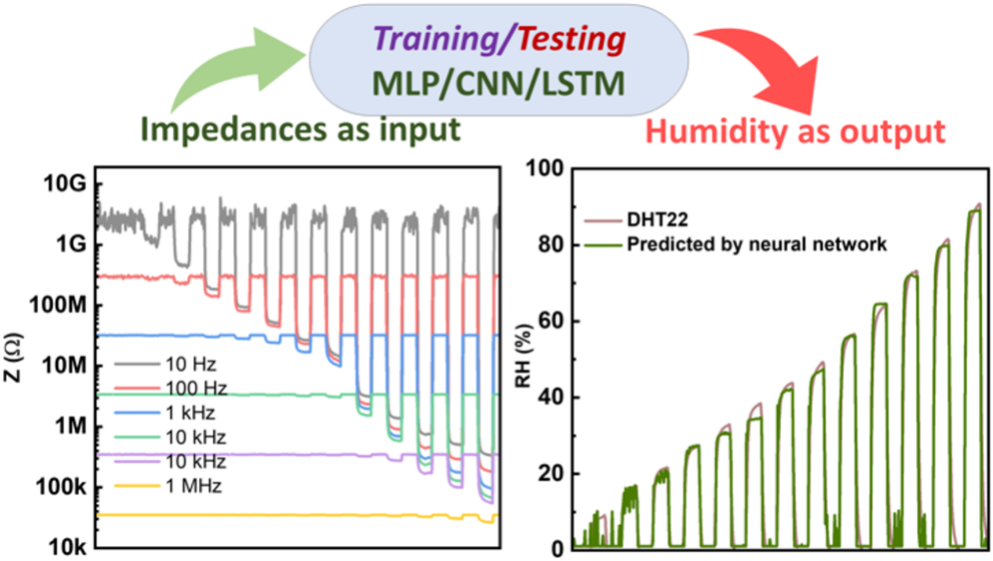

Semiconducting two-dimensional (2D) materials have emerged as promising candidates for gas sensors due to their exceptional sensitivity and rapid response/recovery times. However, these sensors often face significant challenges, including baseline drift, nonlinearity, cross-sensitivity to multiple gases, and early response saturation, all of which compromise their accuracy and reliability. Conventional resistive sensing approaches, which rely on a single output signal for gas concentration estimation, fail to capture the complex interactions inherent to 2D materials, such as charge carrier generation, transport, and polarization. This work addresses these limitations by utilizing impedance measurements across multiple frequencies for MoS_2_- and WS_2_-based sensors, coupled with machine learning-assisted data processing for accurate relative humidity (RH) quantification. By leveraging the impedance domain, we effectively mitigated baseline drift over extended periods and identified mutually exclusive phase behavior for the WS_2_-based sensor. The MoS_2_-based sensor exhibited long-term stability, motivating the application of a neural network-based multilayer perceptron (MLP), one-dimensional convolutional network (1D-CNN), and long short-term memory (LSTM) models to interpret multifrequency impedance data for precise RH measurements. Our approach enabled robust humidity sensing over a wide range (0–90%) with significantly faster response and recovery times than commercial sensors. Additionally, the neural network-assisted WS_2_ sensor effectively minimized cross-sensitivity between humidity and CO_2_. This work showcases the potential of multifrequency impedance-based sensing, combined with machine learning, to overcome the traditional limitations of 2D material-based sensors, offering a pathway toward more reliable, stable, and precise gas-sensing technologies.

Gas sensors are essential tools for the real-time detection of harmful gaseous emissions to ensure the safety of living beings. Although conventional chromatographic and spectral analysis techniques provide better qualitative and quantitative detection, installing them over a large industrial area is hindered by high costs, bulk size and longer response times.^1^ Chemiresistive sensing is a feasible alternative method because of its high sensitivity, rapid detection, and low-cost fabrication.^2−5^ The benefits of miniaturization and integrating multiple sensors on the MEMS platform and simple electrical transduction facilitate the implementation of low-powered wireless nodes for monitoring air quality in industrial areas.^6−9^ However, the practical applications of these sensors are impeded by high susceptibility to cross-interferents and variations in ambient temperature and humidity.^10−13^ The frequently used metal oxide-based chemiresistors require high-temperature operations for efficient detection, leading to baseline and performance drift.^14,15^ Recently, two-dimensional (2D) material-based chemiresistors have emerged as compelling substitutes for metal oxides due to their exceptional electrochemical activity at room temperature.^16−22^ 2D transition metal chalcogenides like MoS_2_ and WS_2_-based materials are potential candidates for detecting many gases, including humidity, NO_2_, H_2_S, NH_3_, and CO, facilitated by their atomically thin layered structure, increased surface area, and inherent defects.^18,23−28^ The greater reactivity of these materials enabled the detection of low gas concentrations; however, the same characteristic leads to response saturation at elevated concentrations. The saturation in response impacts the operational range and yields in the concentration-related nonlinear calibration curve.^29,30^ MoS_2_ and WS_2_ are sensitive to various gases, with a robust response to humidity, which is a common interfering gas in the ambient atmosphere during sensor operation. Identifying incoming gases is challenging due to the substantial impact of humidity on the electrical properties of MoS_2_ and WS_2_-based sensors. Limitations such as performance drift, nonlinearity, response saturation, and the effect of ambient temperature, humidity, and cross-interferents are insignificant in applications such as residential alarms. However, the same drawbacks must be addressed for contemporary applications, such as reliable and precise environmental air quality monitoring and industrial zone surveillance.

Conventional electronic gas sensors are engineered to deliver resistance or current variation as an output in response to variations in gas concentrations.^31^ Controlling and compensating drift by a single DC output is impossible, reducing accuracy and stability.^31^ Chemiresistive measurement at a constant potential is also inadequate for capturing the conduction processes in 2D materials, as resistance is affected by both electronic conduction and ionic diffusion.^23,24^ Electrochemical impedance spectroscopy (EIS) is an effective method for measuring electronic and ionic conductivities, providing a viable alternative to resistive sensing using 2D materials. Many researchers adopted EIS to understand the sensor’s conduction mechanism in different gas atmospheres by analyzing the RC component at high frequencies and a Warburg impedance associated with ion diffusion at low frequencies.^32−35^ Few studies focused on measuring sensor response at a particular frequency for optimal performance.^27,36^ For instance, Potyrailo et al. employed an impedance-based technique to measure linear response to gas-induced variations in the midrange of frequencies and baseline fluctuations at high frequencies.^10,31^ Impedance measurements were conducted at various frequencies for a CuCrO_2_-based composite material to quantify H_2_S while controlling for baseline drift and humidity cross-interference.^37^ In contrast to DC measurements, EIS produces multivariate output across a range of frequencies and aids in capturing various sensor parameters to compensate for baseline drift and temperature/humidity effects.

The present study uses humidity ranging from 0 to ∼90% relative humidity (RH) and CO_2_ to comprehend the behavior of MoS_2_ and WS_2_-based sensors. While water molecules chemisorb and transfer electrons at low humidity conditions, at high humidity, multilayer physical adsorption and Grotthuss mechanism-mediated protonic conduction come into play, facilitating enhanced ion transport.^24,30^ Therefore, impedance-based measurements were systematically examined over a six-decade frequency range to understand these different adsorption and conduction mechanisms in WS_2_ and MoS_2_-based sensors. The excitation frequency was used to control the conduction process and sensor response behavior. The higher response was achieved by operating the sensor at low frequencies. Improved resolution was achieved at higher humidity levels by reducing the saturation through high-frequency measurements. The drift in baseline was successfully controlled by operating a WS_2_-based sensor at high frequencies, and a highly stable baseline was observed for MoS_2_-based sensors. Rapid response and recovery were achieved using a MoS_2_-based sensor, which is better than a commercial DHT22 sensor.

Furthermore, an artificial neural network-based model was utilized to estimate humidity by analyzing various responses generated at different frequencies. The RH was assessed in real-time, demonstrating long-term stability and reproducibility for the MoS_2_-based sensor. Moreover, the signals generated responding to CO_2_ were effectively differentiated by parallelly operating the WS_2_ and the commercial DHT22 humidity sensor. In conclusion, this study provides detailed information on generating multivariate data from 2D material-based sensors, signal processing techniques for precise humidity measurement, and strategies for reducing humidity cross-interference.

## Materials and Methods

### Sensor Fabrication

Microcrystals of WS_2_ and MoS_2_ (99.5% pure, Sigma-Aldrich) were used as precursor materials, and a solution exfoliation method was adopted to fabricate and synthesize the respective nanosheets. 500 mg of precursor materials were sonicated individually using a high-power sonication probe (400 W, Fisher model FB505) in a 250 mL ethanol and water mixture in a 1:1 ratio. Sonication was performed in a pulsed mode with eight-second on–off intervals for 10 h at an amplitude of 80%. The resultant dispersions were centrifuged at 7000 rpm for 10 min. The WS_2_ and MoS_2_ suspensions were collected and used for the subsequent characterizations and sensing experiments.

Scanning electron microscopy (SEM, FEI Magellan 400L) was used to characterize the surface morphology of the sensors, and a thin layer of gold was sputtered to achieve better resolution in the image. The Raman scattering spectra were obtained by using a Horiba T64000 spectrometer with a 532 nm laser line to confirm material quality. TEM (FEI Tecnai G2 F20) was used to confirm the layered structure.

Glass substrates were used for sensor fabrications with Au/Ti (95/5 nm thick, respectively) as the contact material. Laser lithography, e-beam metal deposition, and lift-off procedures were used to fabricate interdigitated electrodes (IDE) with a finger width and interspacing of 25 μm. Figure S1a shows a photograph of the prepared substrate. The 2D material dispersion was drop-cast onto the IDE and dried in an ambient atmosphere.

### Gas Sensor Measurements

The sensors were mounted in a small chamber attached to multiple mass flow connectors (MFCs, Alicat). The gas was passed to the chamber at a constant flow rate of 1000 sccm throughout the sensing experiments. Dry air was used as the carrier gas, and the flow was divided into different ratios and passed through a water bubbler to simulate different humid conditions. The Arduino interfaced DHT22 sensor was operated continuously inside the sensing chamber to measure the relative humidity (RH) and temperature variation. The RH varied from 0 to ∼95%, and the room temperature varied from 18 to 42 °C. To explore the electrical characteristics of the sensors and examine the effects of gas adsorption/desorption and the resulting charge transport mechanism, electrochemical impedance spectroscopy (EIS) was carried out. Hameg 8118 was used for preliminary impedance measurements, and Analog Discovery 2 was used in later stages. Impedance was collected from 10 Hz to 1 MHz with a sinusoidal voltage of 0.2 V. Frequency range and sinusoidal voltage were chosen to achieve optimal response and lower noise under humidity conditions. The schematic representation of the experimental setup is shown in Figure S1b.

### Neural Network

Neural network-based analysis was conducted using Python as the programming language. The computer equipped with 32GB RAM and Intel Xeon Processor E5-1620 v4 was used for analysis. Multilayer perceptron (MLP), one-dimensional convolutional neural network (1D-CNN), and long short-term memory (LSTM) networks were chosen to map impedance-domain data in different humid atmospheres to the estimated RH value using a DHT22 sensor. The neural networks were implemented and tuned in a TensorFlow environment using libraries such as Keras for neural network construction, NumPy for data manipulation, and Scikit-learn for preprocessing the data. Standardization was employed as a preprocessing step to set the mean of six impedance values associated with a particular humid condition to zero with a standard deviation of one. The neural networks were trained for multiple epochs using sensor data, and checkpoints were saved at a frequency of 20 epochs. The hyperparameters were manually tuned to improve the model performances. Test data was used to assess these checkpoints’ performance, and the best-performing model was chosen for the sensor’s long-term assessment and CO_2_ detection.

## Results and Discussion

**Figure 1:**
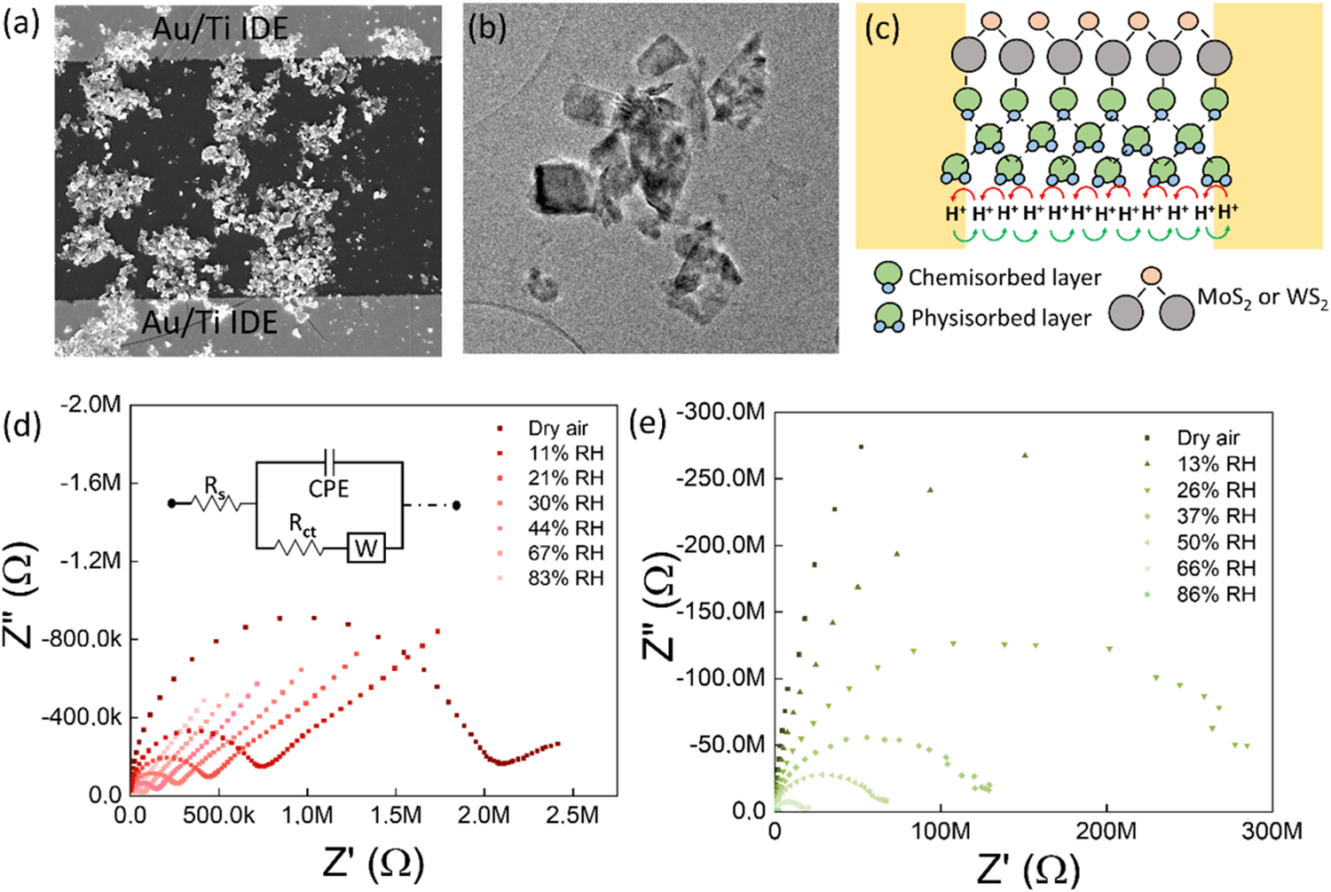
(a) Representative SEM image of 2D material coated Au/Ti IDE, (b) TEM image of WS_2_, and (c) schematic representation of conduction in 2D materials. (d) Nyquist plot for WS_2_-based sensor in 0 to ∼83% RH. (e) Nyquist plot for MoS_2_-based sensor in 0 to ∼86% RH.

The morphology obtained from drop-casting consisted of interconnected few-layer 2D material flakes, as shown in SEM and TEM Figure 1a,b. The interconnected MoS_2_ and WS_2_ flakes were highly crystalline, as evidenced by the high-resolution TEM images in Figure S2. Additionally, they possess a significant surface-to-volume ratio and a considerable number of reactive edges. The IDEs were connected by a complex network of semiconducting 2D material flakes with multiple material-electrode interfaces. The EIS results for this morphology in dry air demonstrated a decrease in impedance and an −90° shift in phase difference with the increase in excitation frequency, as illustrated in Figure S3. This relaxation behavior may be attributed to the dynamic interactions among charge carriers in the 2D material flakes and the IDEs on the substrate. This behavior resembled a combination of parallel RC circuits, wherein the IDE denotes the capacitive elements with multiple interfaces, and the resistive elements are characterized by a dielectric glass substrate coated with 2D material. The impedances of both WS_2_ and MoS_2_ decreased with increasing humidity, as demonstrated in Figure S3. The observed behavior can be attributed to the interaction of H_2_O molecules with the 2D material surface. To understand the interaction better, the real and imaginary impedance components were calculated and displayed as Nyquist plots, as shown in Figure 1d,e for the WS_2_ and MoS_2_-based sensors, respectively. At lower frequencies, a linear behavior region was observed, while a semicircle appeared at higher frequencies, which validated the presence of multiple conduction mechanisms in both sensors at high humidity levels. The linear behavior confirmed the diffusion of ionic species toward the electrodes, represented as a Warburg component (*W*) in the equivalent circuit model.^32,33,38^ At higher frequencies, diffusion was restricted, and conduction through generated charge carriers became dominant, producing the semicircle associated with charge transfer. The linear behavior was absent in dry air and at lower humidity for the MoS_2_-based sensor (Figure 1e), indicating the absence of diffusion under these conditions. In contrast, the shorter tail observed in the dry-air environment for the WS_2_-based sensor supports the presence of diffusive ionic species (Figure 1d), even after drying for more than an hour at ambient temperature. This could be attributed to the presence of strongly adsorbed water molecules within the layered structure. The impedance of the WS_2_-based sensor progressively increased over several hours, leading to the less pronounced effect from the Warburg element associated with diffusion (as shown in Figure S4).

The sensing mechanism described above confirms the chemisorption of water molecules onto the material surface and resistance reduction by donating electrons under lower humidity.^23,27^ This process is promoted by a larger surface area characterized by numerous edge sites and defects^27,29^ (Figures S5 and S6, Note 1, Supporting Information). The absence of a linear behavior region associated with ionic diffusion suggested predominant chemisorption and electronic conduction at lower humidity. Water molecules physically adsorb on the chemisorbed layer with increased humidity,^30^ as illustrated in Figure 1c. Multilayered physically adsorbed water molecules can self-ionize (H_2_O ⇌ H^+^ + OH^–^) on the surface of 2D materials, resulting in the formation of protons (H+) and hydroxide ions (OH^–^).^27^ The produced H+ ions protonate the closest water molecule (H_2_O + H^+^ ⇌ H_3_O^+^), forming hydronium ions (H_3_O^+^).^27^ The protons can hop from hydronium ions to adjacent water molecules in an electric field, replicating the conduction process observed in bulk liquid water (H_2_O + H_3_O^+^ → H_3_O+ + H_2_O).^27,38^ The diffusive element observed in the Nyquist plot at elevated humidity levels can be attributed to protonic conduction in 2D materials, and the Grotthuss mechanism serves as a framework for understanding the ionic conduction in two-dimensional materials.^16,33^

**Figure 2:**
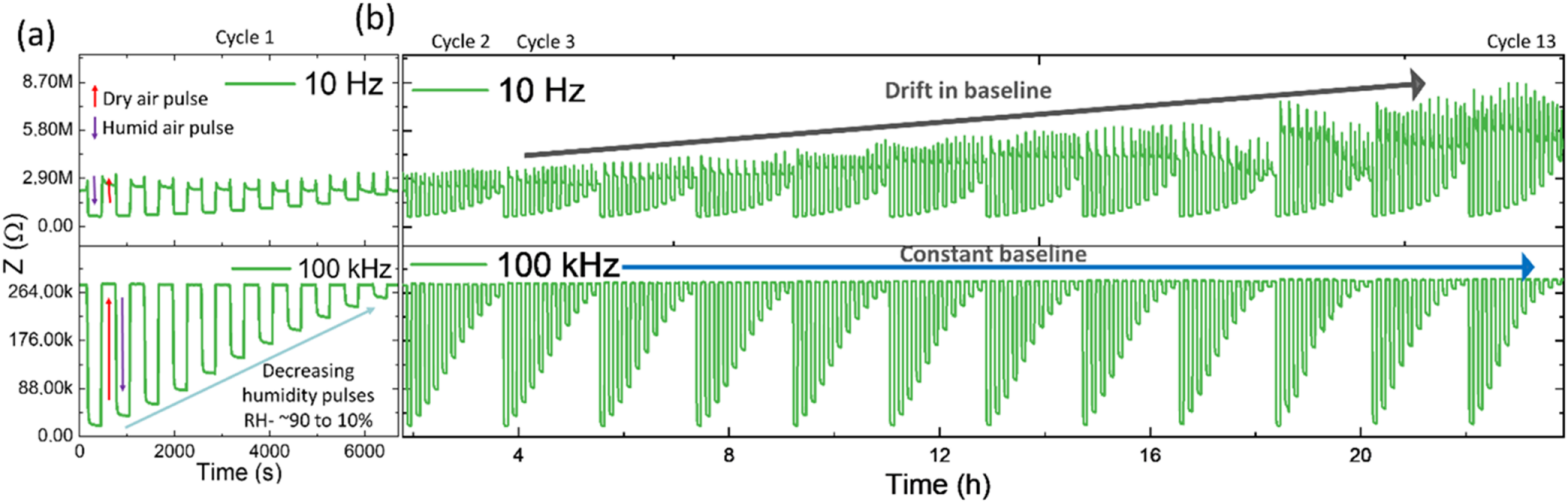
Impedance variations of the WS_2_ sensor collected at 10 Hz and 100 kHz at different humidity levels. The experiment was conducted over 13 cycles with varying humidity levels. The first cycle is magnified and presented in (a), while (b) illustrates the drift in the baseline.

**Figure 3:**
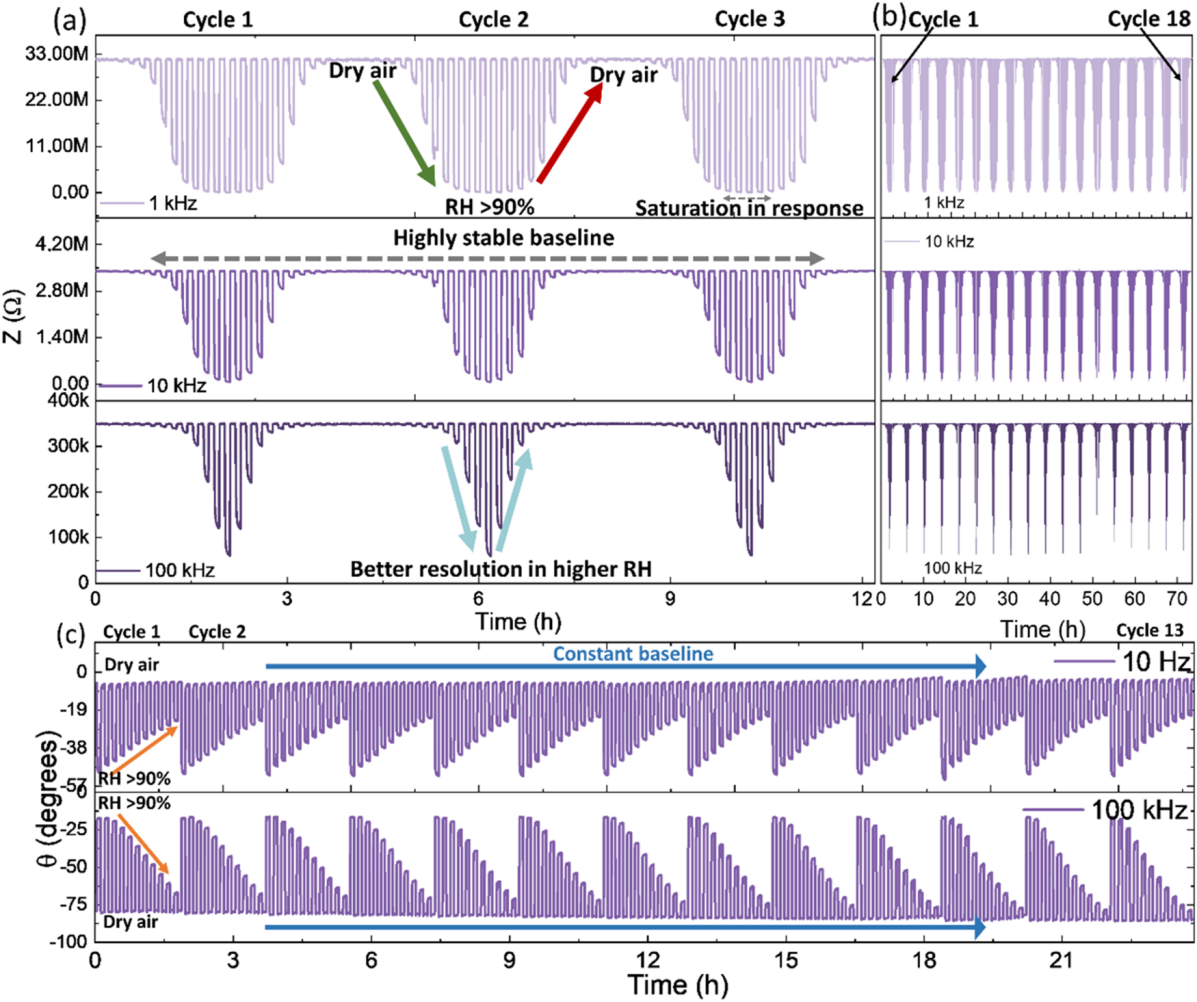
(a) Impedance variation collected for the MoS_2_-based sensor at varying humidity, and (b) long-term reproducibility and stability observed for the MoS_2_-based sensor. The data were collected over 18 repeated cycles of varying humidity for ∼72 h, with the first three cycles magnified and illustrated in (a). (c) The variation of phase difference collected for the WS_2_-based sensor at different humidity levels. The experiment was repeated for 13 cycles, varying humidity, to observe drift in the baseline.

The observed gradual decrease in charge transfer resistance and increase in diffusion with increasing humidity suggested that operating the sensor at only one frequency is insufficient to capture its complete characteristics. As a result, impedance-based data at multiple frequencies over time were systematically collected under different RHs, as shown in Figures 2 and 3. Impedance data showed that both sensors responded strongly to RH variation at lower frequencies due to electronic and ionic conduction. The absence of diffusion in both sensors possibly reduced the response at high frequencies. The attenuated impedance variation at higher frequencies facilitated enhanced resolution at increased RH concentrations by preventing response saturation, as shown in Figures 2 and 3a.

The gradual drift in the baseline was observed during the continuous operation of the WS_2_-based sensor at 10 Hz, as shown in Figure 2. In contrast, drift was negligible for data collected above 100 kHz. The sensor performance over the complete frequency range can be seen in Figure S7. As seen in the Nyquist plots in Figures 1d and S4, the decrease in the Warburg component indicates the gradual desorption of diffusive ionic species from the layered structure, possibly responsible for baseline drift at low frequencies. In contrast, desorption did not affect the RC components at high frequencies and resulted in a stable baseline. The performance of the WS_2_ sensor degraded after a prolonged operation lasting up to a week, as shown in Figure S8. This can be attributed to continuous operation in a humid environment, which may have reduced the number of reactive adsorption sites in WS_2_ flakes. As illustrated in Figures 1d and S4, the observed increase in charge transfer resistance indicates possible oxidation at the edges, which may create a barrier and result in a decreased level of performance and baseline drift.

For the MoS_2_-based sensor, the impedance in dry air was extremely high, resulting in noisy measurements at low frequencies, as shown in Figure S9. Very high response of 13,800 was observed for 85% RH at 10 Hz and 2400 at 100 Hz, and the calculated response is shown in Figure S10. The sensor baseline was highly stable with a reproducible response at higher frequencies (as shown in Figure 3a,3b), demonstrating the reliability of the MoS_2_. Consequently, the MoS_2_-based sensor was operated for a month, and data were collected on various days. The collected data revealed the MoS_2_-based sensor’s exceptional repeatability.

Figure 3c illustrates the variation of phase differences for WS_2_-based sensors. Increasing humidity produced contradictory trends; an increase in phase difference was noticed at 10 Hz, while a decreasing trend was apparent at 100 kHz. The larger phase difference at low frequencies can be attributed to the dominance of diffusion processes. In contrast, the reduction in the phase difference showed the dominance of the sensor’s RC components at high frequencies. This conflicting behavior can be effectively utilized to enhance the efficacy of humidity monitoring systems in real-world applications. The sensor performance over the complete frequency range can be seen in Figure S11. The contradictory behavior was not observed in MoS_2_-based sensors, most likely due to higher impedance, as shown in Figure S12. In contrast to the impedance data, a consistent baseline in phase difference was observed at 10 Hz for the WS_2_-based sensor, as shown in Figure 3c. This behavior could be because of restricted ionic mobility in a dry-air atmosphere. A constant baseline of approximately −90° was observed at 100 kHz, indicating the dominance of capacitive elements in a dry-air atmosphere.

**Figure 4:**
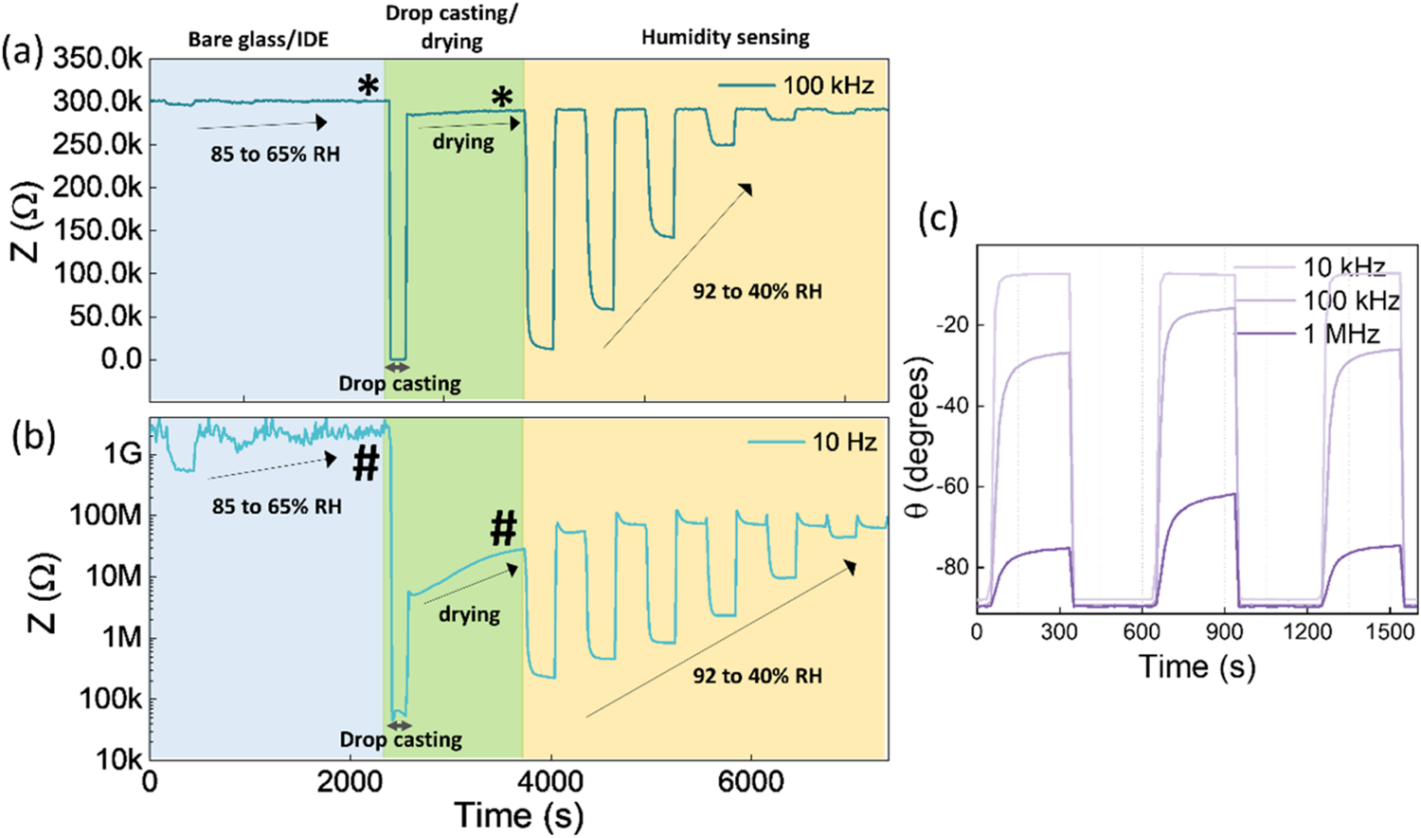
Impedance monitoring at (a) 100 kHz and (b) 10 Hz during the MoS_2_ sensor fabrication process. The humidity sensing behavior of bare glass substrate is highlighted in blue, impedance variation during drop-casting and drying is highlighted in green, and subsequent humidity sensing is highlighted in yellow, respectively. (c) Comparison of response and recovery behavior at different frequencies in phase domain.

Continuous impedance tracking during different fabrication stages helped us to understand the contributions of the substrate, IDE, and active sensing materials. Specifically, the sensor impedance was monitored at three critical stages of fabrication: before deposition (IDE/substrate), during drop-casting of MoS_2_ dispersion on the IDE, and throughout drying. Humidity sensing experiments conducted on an IDE-coated bare glass substrate before the drop-casting of MoS_2_ demonstrated a poor response; for example, the response measured at 100 kHz is shown in Figure 4a (highlighted in blue). Afterward, the impedance decreased during drop-casting due to the presence of solvent molecules in the MoS_2_ dispersion, and the evaporation of these molecules increased the impedance. The measured impedance during the coating and drying process is illustrated in Figure 4a (highlighted in green). As indicated by asterisks in Figure 4a, similar impedance was observed in dry air before and after MoS_2_ coating, which implied negligible contribution from the coated material at higher frequencies. A significant impedance variation can be observed in the subsequent humidity sensing characterization carried out during the fabrication process, as shown in Figure 4a (highlighted in yellow). This demonstrates that the impedance measured in a humid atmosphere originates from the humidity interaction with MoS_2,_ and the baseline impedance in dry air comes from the substrate IDE structure.

The impedance measured at 10 Hz during the fabrication process is shown in Figure 4b. Very high impedance was observed at 10 Hz for the bare glass IDE structure and was substantially decreased by the MoS_2_ coating, as indicated using “#” in Figure 4b. Solvent evaporation resulted in a slower increase in impedance as compared to measurements done at 100 kHz, as highlighted in green in Figure 4b. Subsequent humidity sensing experiments revealed a drift in the baseline (Figure 4b, highlighted in yellow) because of the gradual desorption of ionic species.

The MoS_2_-based sensor also showed a faster response and recovery. Figure 4c shows the response and recovery behavior in the phase domain at different frequencies. Figure S13 compares the impedance-domain behavior at various frequencies. Furthermore, Figure S14 shows that MoS_2_-based sensors operated at 10 kHz attained steady saturation impedance much faster than the DHT22 sensor. The fast response and recovery can be attributed to the absence of a slower diffusive process at 10 kHz.

### Neural Network-Based Analysis

**Figure 5:**
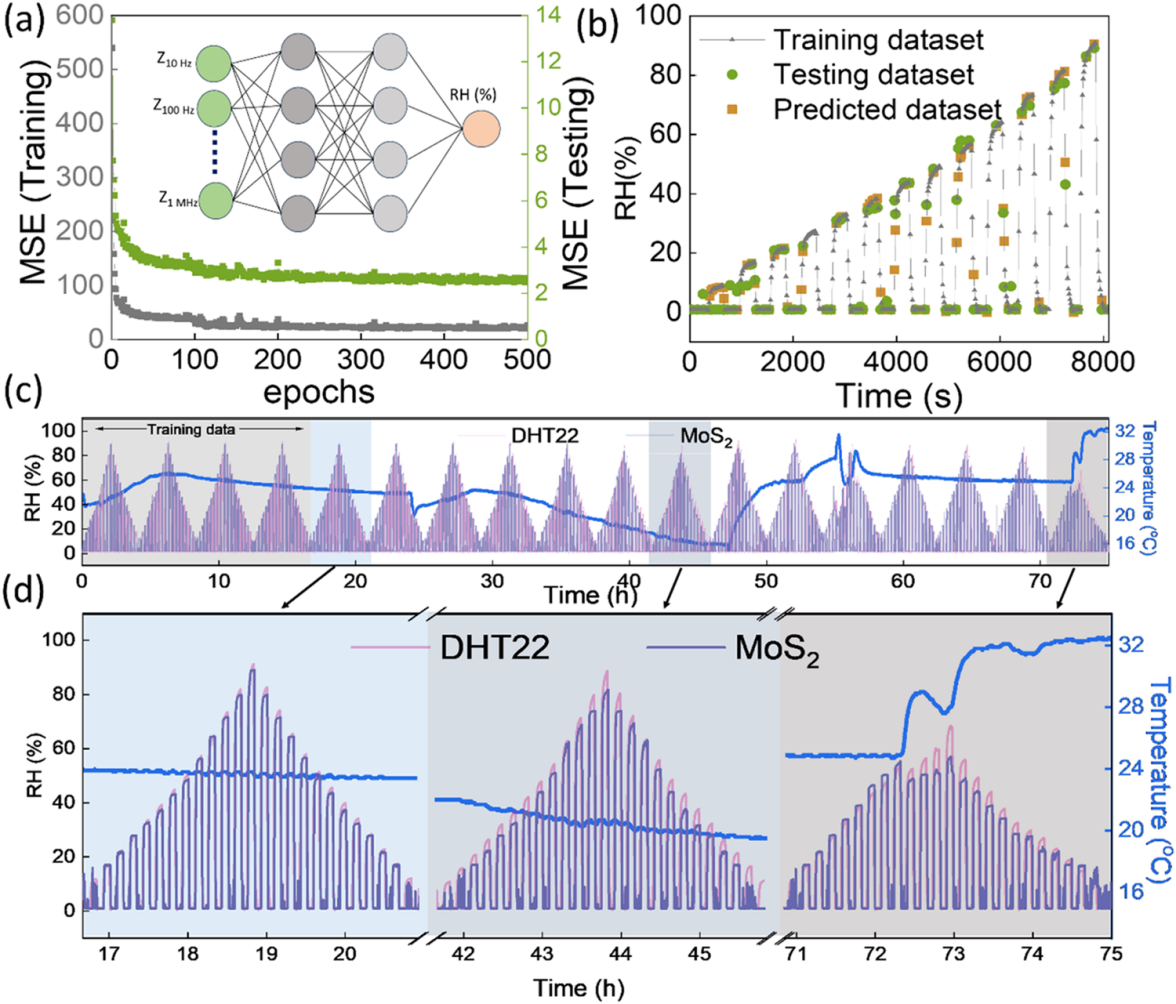
(a) Reduction in MSE with respect to epochs, schematic representation of MLP can be seen in the inset, and (b) typical data distribution used to train MLP and subsequent predicted data points. (c) Predicted data for an extended duration using an MLP-based model. (d) Magnified image of estimated humidity values at different intervals. The temperature fluctuations within the sensing chamber during the measurements are also illustrated in (c, d).

Artificial neural network-based algorithms such as MLP, 1D-CNN, and LSTM models were adopted to estimate the RH by analyzing the multivariate impedance-based data. The MoS_2_-based sensor was chosen to train the predictive model because of its long-term stability, as reported in our previous study.^24^ The MLP was constructed with six nodes in the input layer to receive six impedance values measured at different frequencies. The six hidden layers were configured with 20, 100, 500, 1000, 500, and 20 nodes, respectively, and a single node was used in the output layer to estimate the humidity. The inset of Figure 5a illustrates the schematic architecture of MLP, and a model summary is given in Figure S15. To ensure MoS_2_-based sensor reliability, DHT22 was operated in parallel to measure real-time RH and room temperature. MLP was trained to predict the RH estimated using a DHT22 sensor for each set of impedance-based input data collected at six different frequencies. The training was iterated over 500 epochs to update the network parameters using adaptive moment estimation (Adam) as the optimizer, the rectified linear unit (ReLU) as the activation function, and mean absolute error (MAE) as the error function.

**Figure 6:**
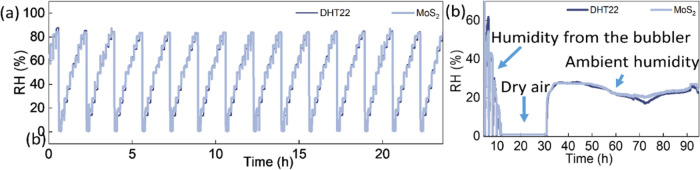
(a) Prediction output of MLP in comparison with commercial sensor DHT22 after one month of operation and storage. (b) Measuring ambient humidity using a MoS_2_-based sensor after one month of operation.

Various data cycles in different humidity conditions were collected for nearly 3 days. The MLP was trained and tested using the initial four cycles and validated for the subsequent 14 cycles, as highlighted in Figure S16. The decrease in mean squared error (MSE) between predicted and actual value with respect to the epoch is shown in Figure 5a. The typical DHT22 data distribution used for the training and testing, as well as the estimated RH values for the test data, is shown in Figure 5b. The room temperature was varied from 16 to 42 °C using an external cooler and heater to ensure the generalizability and robustness of the model and the MoS_2_-based sensor, respectively. The MLP-assisted MoS_2_ sensor successfully measured the humidity in a variable temperature environment, as shown in Figure 5c,d. Even though the MoS_2_-based sensor produced a nonlinear response at all the measured frequencies, as shown in Figure S17, the MLP-based model successfully mitigated the calibration difficulty that arose from such nonlinearity.The data were acquired for various days and analyzed using the finalized MLP model to ensure repeatability. After a month of operation, the estimated RH values are shown in Figure 6a, and reproducibility tests performed on different days are presented in Figures S18–S20, proving the robust performance of the MoS_2_-based sensor. Further, experiments were extended to estimate RH in an ambient room atmosphere. Impedance-based data for MoS_2_-based sensors were gathered continuously in a typical laboratory atmosphere and evaluated using the pretrained MLP model. The MoS_2_-based sensor-assisted MLP model accurately determined indoor RH, as shown in Figure 6b (Note 3, Supporting Information). The model architecture, learning curve, and predicted data related to 1D-CNN and LSTM are shown in Figures S21–S26.

**Figure 7:**
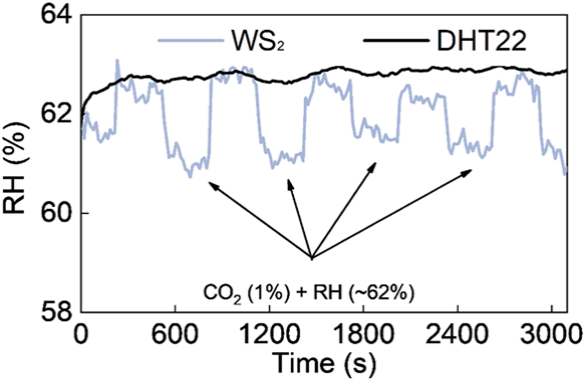
Deviation in humidity estimation by the WS_2_-based sensor in the presence of CO_2_.

Finally, successful humidity estimation has prompted the development of a concept to detect cross-interference produced by additional gas in the presence of humidity. Both MoS_2_ and WS_2_ sensors were characterized to detect CO_2_ (1%) in the presence of humidity. The impedance of the MoS_2_-based sensor remained stable during CO_2_ exposure, indicating notable selectivity for humidity. As illustrated in Figure S27, a minor impedance increase was noted for the WS_2_ sensor in the CO_2_ atmosphere. WS_2_ has higher carrier concentration and superior carrier mobility, which could contribute to an increased reactivity and selectivity to CO_2_ compared to MoS_2_.^39^ So, the WS_2_-based sensor was employed to tackle the issue of cross-sensitivity toward CO_2_. Initially, an MLP-based model was trained to estimate the RH in real-time from the WS_2_-based sensor data. The MLP architecture and training procedure were the same as described previously. When WS_2_-based and DHT22 sensors operated simultaneously in a humid environment, both sensors accurately estimated the humidity. With exposure to CO_2_, the humidity estimated from the WS_2_ sensor deviated from that of the DHT22 sensor. The increase in impedance with CO_2_ exposure compelled the WS_2_ sensor-assisted MLP model to estimate the decreased humidity. The DHT22 sensor served as a reference sensor for accurate humidity estimation in the mixture, and the deviated signal from the WS_2_-based sensor represented cross-interference from CO_2_. Figure 7 depicts the variation in estimated RH from the DHT22 and WS_2_-based sensor in the presence of a CO_2_ and humidity mixture. The difference in RH estimation from both sensors can be used to quantify the concentration of CO_2_.

In conclusion, the impedance measurement-assisted 2D material-based sensors provide various advantages over the traditional chemiresistive sensors. First of all, the 2D flake network morphology enhances the sensor performance and minimizes material consumption compared to conventional thick and continuous film-based gas sensors. This approach also allows for further miniaturization and the development of MEMS-based platforms using AC electrical signal transduction. The impedance-based methodology facilitated the quantification of the response produced from different conduction mechanisms in 2D material-based sensors, a capability not provided by DC resistive methods. High-frequency electrical excitation is efficiently utilized to address the prevalent issue of response saturation associated with 2D materials and DC measurements. This method efficiently controls the baseline drift by tuning the operation frequency, a feature absent in the DC resistive methodology. Moreover, it significantly reduces response and recovery times with exceptional stability compared with commercial sensors, potentially increasing the efficiency of humidity sensors. The proposed signal processing technique addressed cross-sensitivity concerns, improving the selectivity within the 2D material-based sensor system.

## Conclusions

We have developed an impedance-based sophisticated gas-sensing methodology for semiconducting 2D material-based humidity sensors. This innovative approach delivers an adaptive response across a wide concentration range of test gases and maintains baseline stability while significantly mitigating sensor saturation. This also offers multiple outputs corresponding to different phenomena in 2D materials for precise quantification of the test gas. Long-term evaluation of WS_2_-based sensors showed degradation in performance, while MoS_2_-based sensors remained stable for more than a month. We successfully captured the oppositely behaving signal in a phase domain for a WS_2_-based sensor corresponding to polarization and charge carrier-related relaxation process. Furthermore, we successfully showed high baseline stability for both sensors in the high-frequency range. A neural network-assisted multivariate analysis model was also built to quantify the humidity by providing nonlinear responses at multiple frequencies as input. The performance of the MoS_2_-based sensor was comparable to that of the commercially available DHT22 humidity and temperature sensor in both test and ambient atmospheres, with improved response and recovery times. The humidity estimation in the ambient atmosphere by the 2D material-based sensor can help to operate the sensor efficiently in cross-sensitive environments. The CO_2_ was effectively detected in the presence of humidity by operating a WS_2_-based sensor and a DHT22 sensor parallelly. Our findings provide a solid foundation for future innovations in gas sensor technology and pave the way for robust, low-cost sensor array development that can be integrated into wireless networks for enhanced environmental monitoring. This approach holds promise for broad adoption in numerous applications, ranging from industrial safety to environmental conservation, significantly impacting public health and safety.

## Supporting Information Available

Experimental schematic diagram, TEM image, impedance and phase spectra, Raman spectra and analysis of MoS_2_ and WS_2_, sensor response measurements, model summary of neural networks, long-term sensor evaluation results (PDF)
