# Supplementary material for: Impedance-Assisted Multivariate Analysis Technique for Enhanced Gas Sensing with 2D Dichalcogenides
Source: ACS Sens. 2025 Mar 31;10(4):2712–20. doi: 10.1021/acssensors.4c03325 (PMC12038879; doi:10.1021/acssensors.4c03325)
Supplement: Supplementary file 1 — se4c03325_si_001.pdf [file se4c03325_si_001.pdf]

Supporting Information

Impedance-assisted multivariate analysis technique for enhanced gas sensing with 2D dichalcogenides

Bharath Somalapura Prakasha [a], Peng Xiao [a], María José Esplandiu [a], JiaQi Yang [a], Daniel Navarro-Urrios [b], Javier Rodríguez-Viejo [a,c] and Marianna Sledzinska,[a*]*

[a] *Catalan Institute of Nanoscience and Nanotechnology (ICN2), CSIC and BIST, Campus UAB, Bellaterra, Barcelona 08193, Spain*

[b] *MIND-IN2UB, Departament d'Enginyeria Electrónica i Biomédica, Facultat de Física, Universitat de Barcelona, Martí i Franquès 1, Barcelona 08028, Spain*

[c] *Departament de Fisica, Facultat de Ciencies, Universitat Autonoma de Barcelona, Bellaterra, Barcelona 08193, Spain.*

* Email: bharath.somalapura@icn2.cat, marianna.sledzinska@icn2.cat

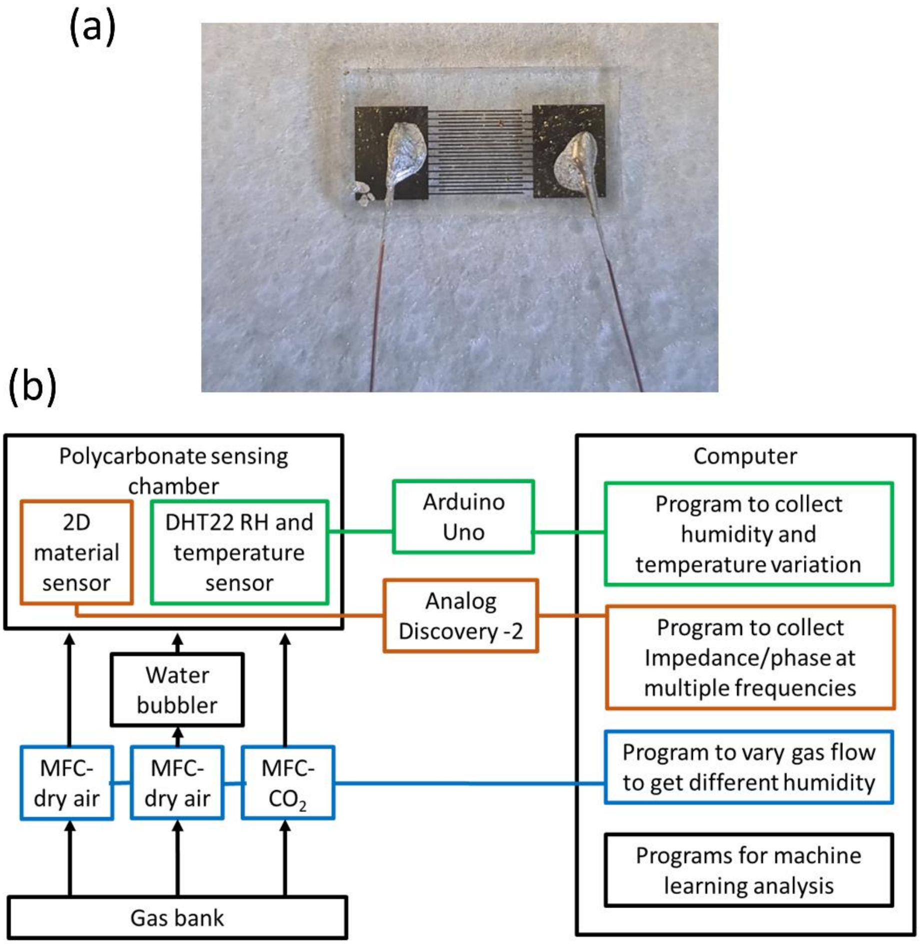

Figure S1. (a)Photograph of IDE-coated glass substrate attached with copper wires, (b) schematic representation of sensing experimental setup.

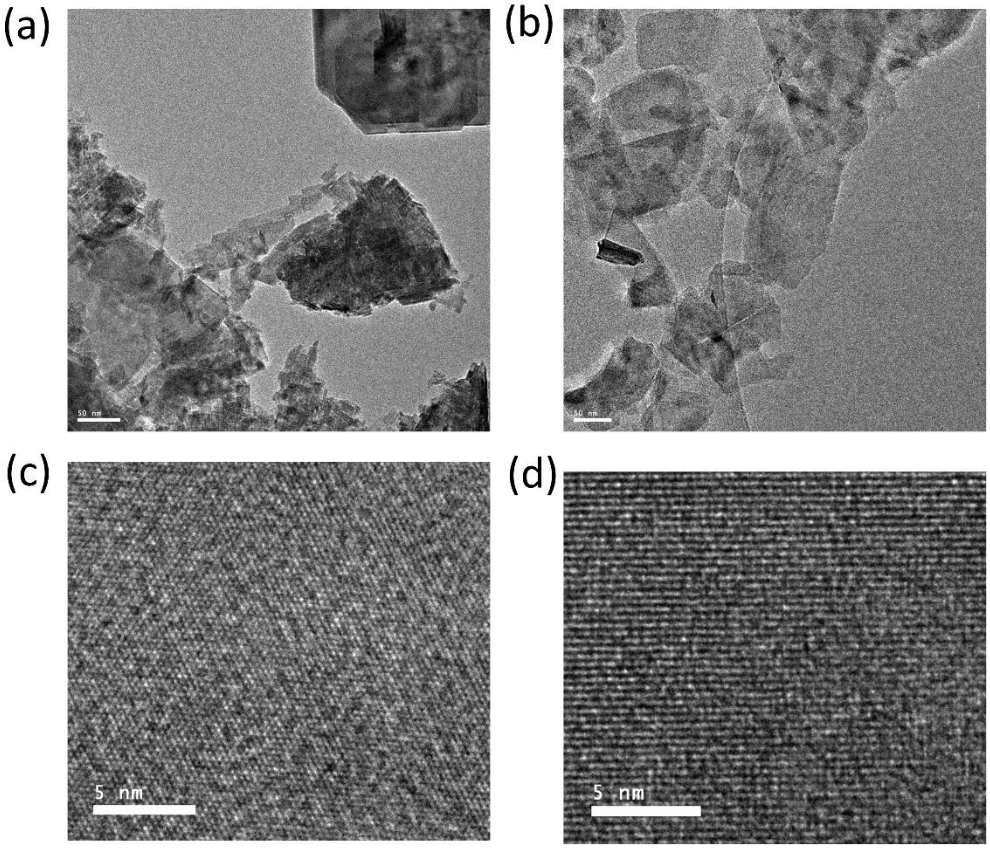

Figure S2. TEM image of (a) WS_2, (b) MoS_2, (c) magnified image of WS_2, and (d) magnified image of MoS_2

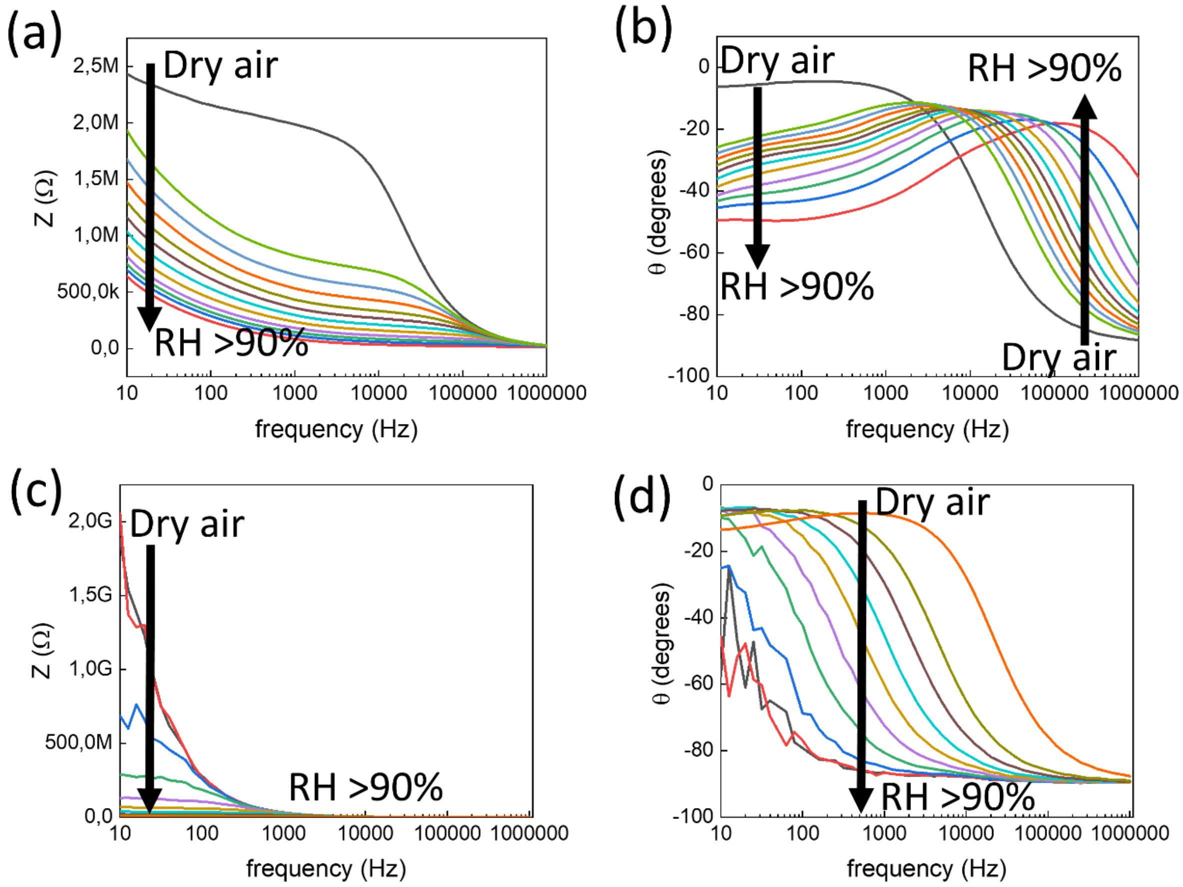

Figure S3. (a) And (b) impedance and phase spectra in different humid atmospheres for WS_2-based sensors. (c) And (d) impedance and phase spectra in different humid atmospheres for MoS_2-based sensors.

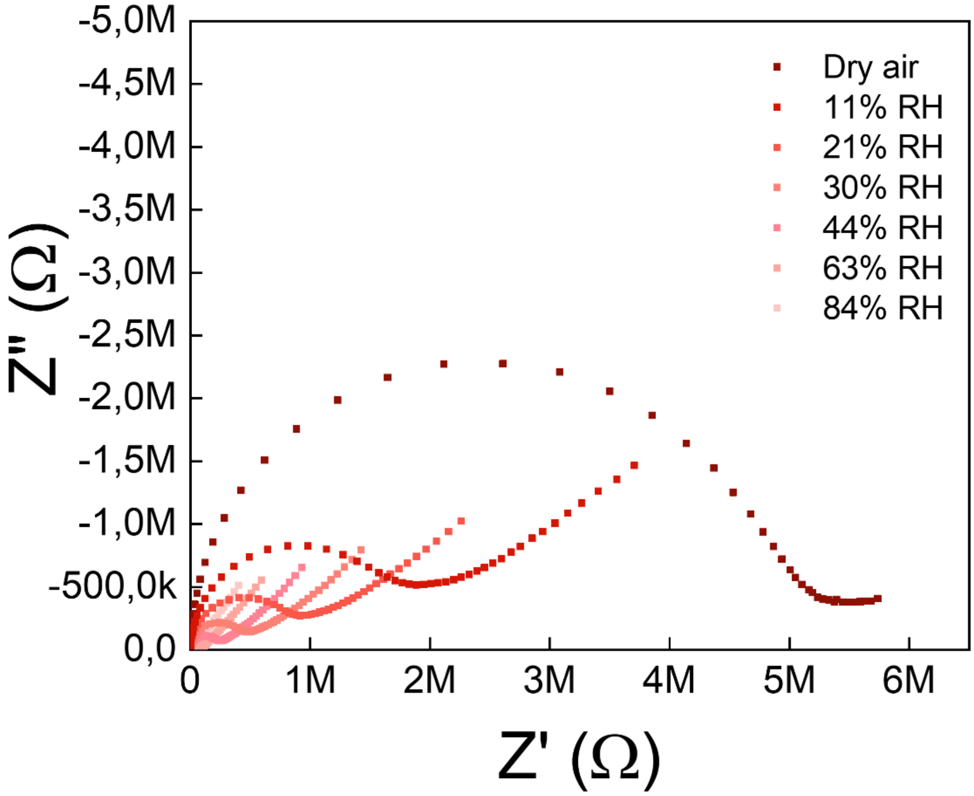

Figure S4. Nyquist plot for WS_2-based sensor in 0 to ~84% RH after 24h of operation

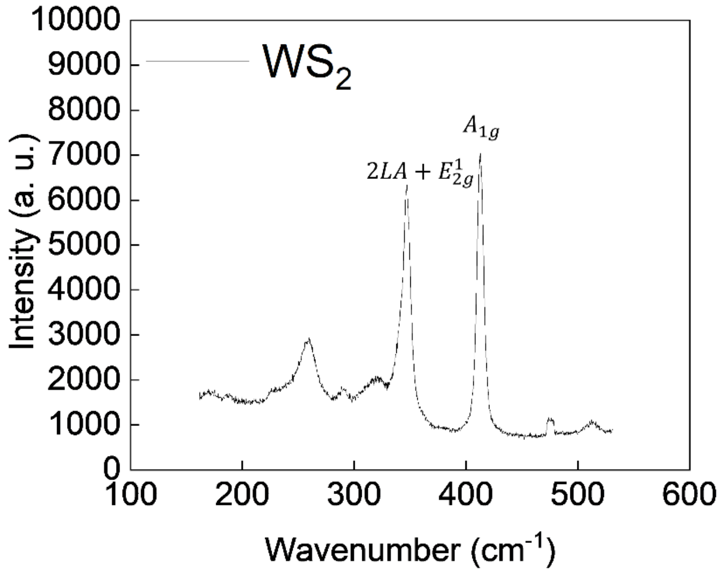

Figure S5. Raman spectra of WS_2-based sensor

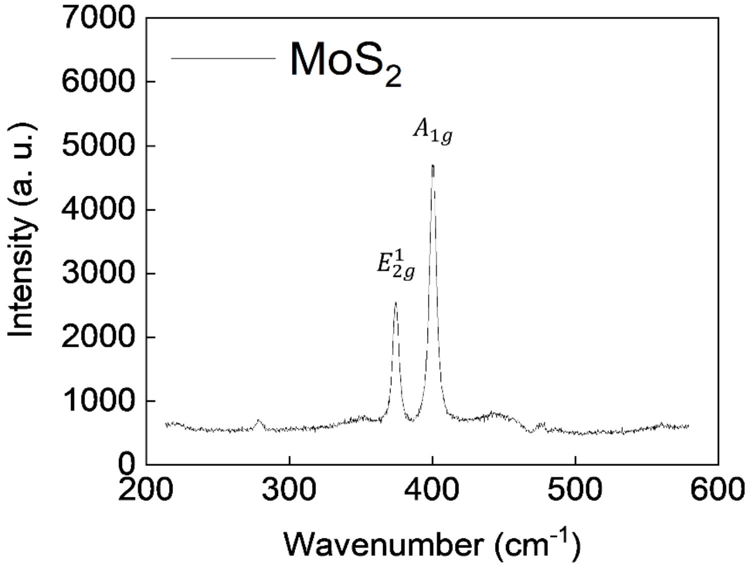

Figure S6. Raman spectra of MoS_2-based sensor

Note 1:

Figure S5 shows the Raman spectra of flakes from our WS_2 and MoS_2-based samples (with soda-lime glass as the substrate). Two distinct vibrational modes, E_{2g}^1 and A_{1g}, are clearly observed for WS_2 and MoS_2. The E_{2g}^1 mode arises from the in-plane vibrations of sulphur atoms relative to a molybdenum or tungsten atom. In contrast, the A_{1g} mode is associated with the out-of-plane vibrations of sulfur atoms [1]. The measured E_{2g}^1 peak is at 374.43 cm^{-1} for MoS_2 and 384.49 cm^{-1} for WS_2, respectively. The A_{1g} peaks appear at 400.50 cm^{-1} for MoS_2 and 412.73 cm^{-1} for WS_2. The separation between the E_{2g}^1 and A_{1g} peaks for both materials indicate the presence of multiple layers. Additionally, the narrow and sharp A_{1g} peaks suggest the samples have high crystalline quality.

[1] K. Golasa, R.Bozek M.Grzeszczyk, A.Wysmolek P.Leszczynski, A.Babinski M. Potemski, Resonant Raman scattering in MoS2-From bulk to monolayer, Solid State Commun 197 (2014) 53–56.

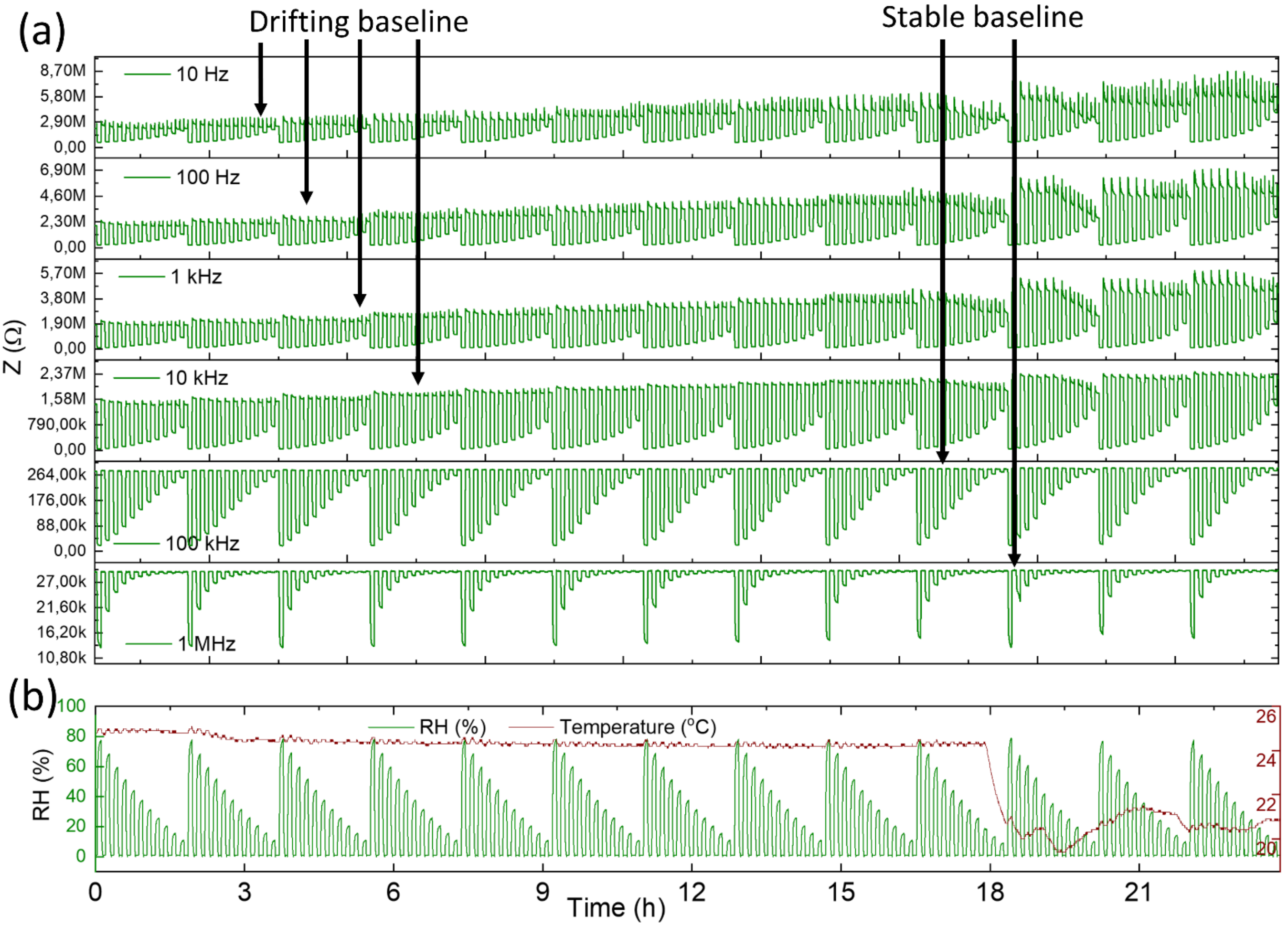

Figure S7. (a) Comparison of WS_2-based sensor impedance behaviour at various frequencies; (b) Humidity estimated by DHT22 sensor. A WS_2-based and DHT22 sensors were operated in parallel to observe humidity variation in the chamber. The 13 cycles of varying humidity levels (0-80% RH) passed through the sensing chamber to monitor baseline drift.

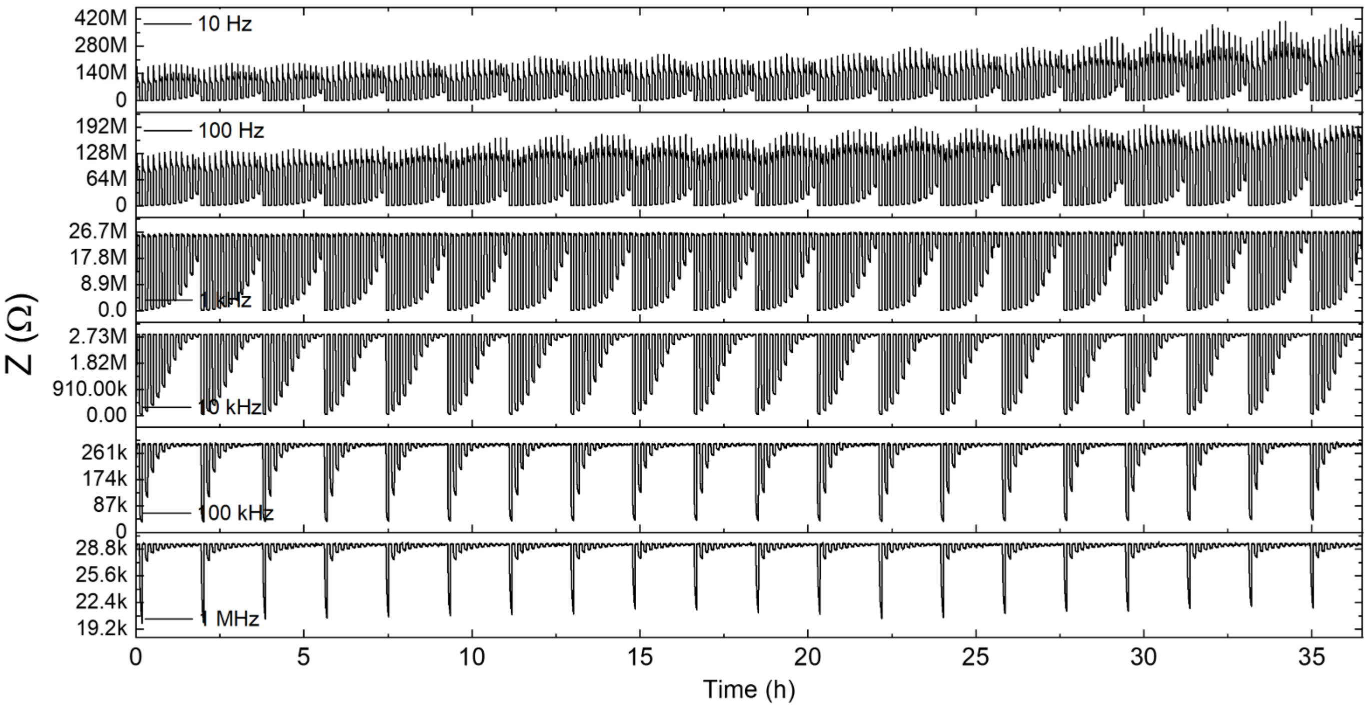

Figure S8. Impedance values collected for WS_2-based sensors at different humidity conditions after one week of operation. The decrease in performance can be observed in comparison with the data given in Figure S7.

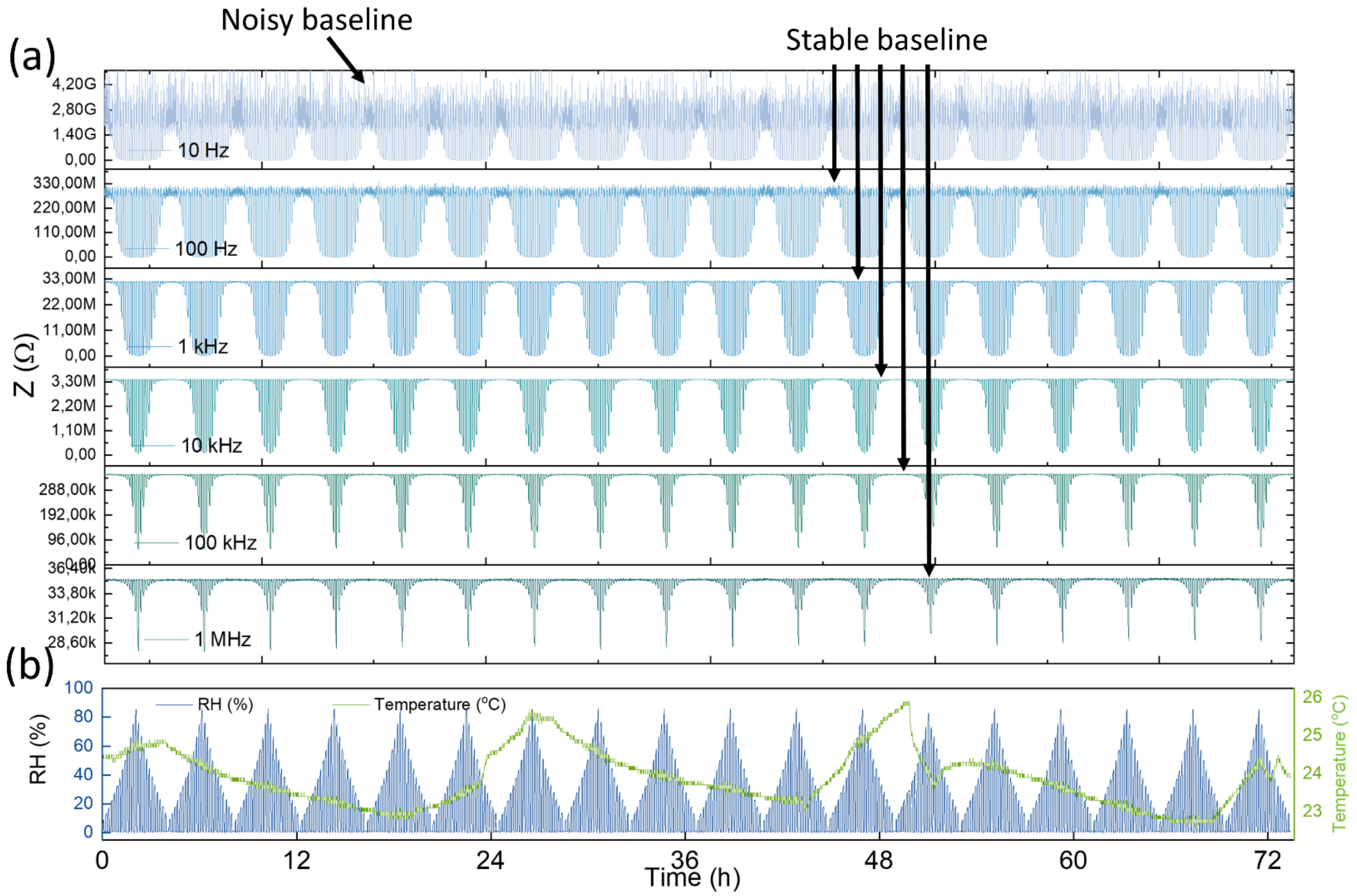

Figure S9. (a) Comparison of MoS_2-based sensor impedance behaviour at various frequencies; (b) Humidity estimated by DHT22 sensor. The MoS_2-based and DHT22 sensors were operated in parallel to observe humidity variation in the chamber. The 18 cycles of varying humidity levels (0-90% RH) passed through the sensing chamber to monitor baseline drift.

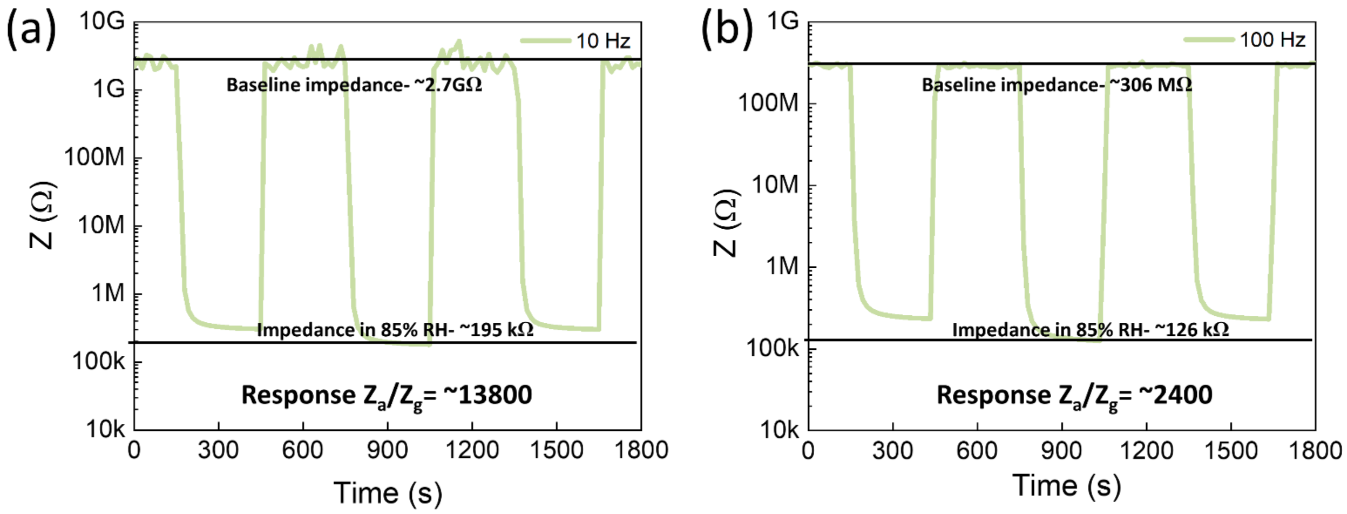

Figure S10. Demonstration of response calculation for MoS_2-based sensor at (a) 10 Hz and (b) 100 Hz.

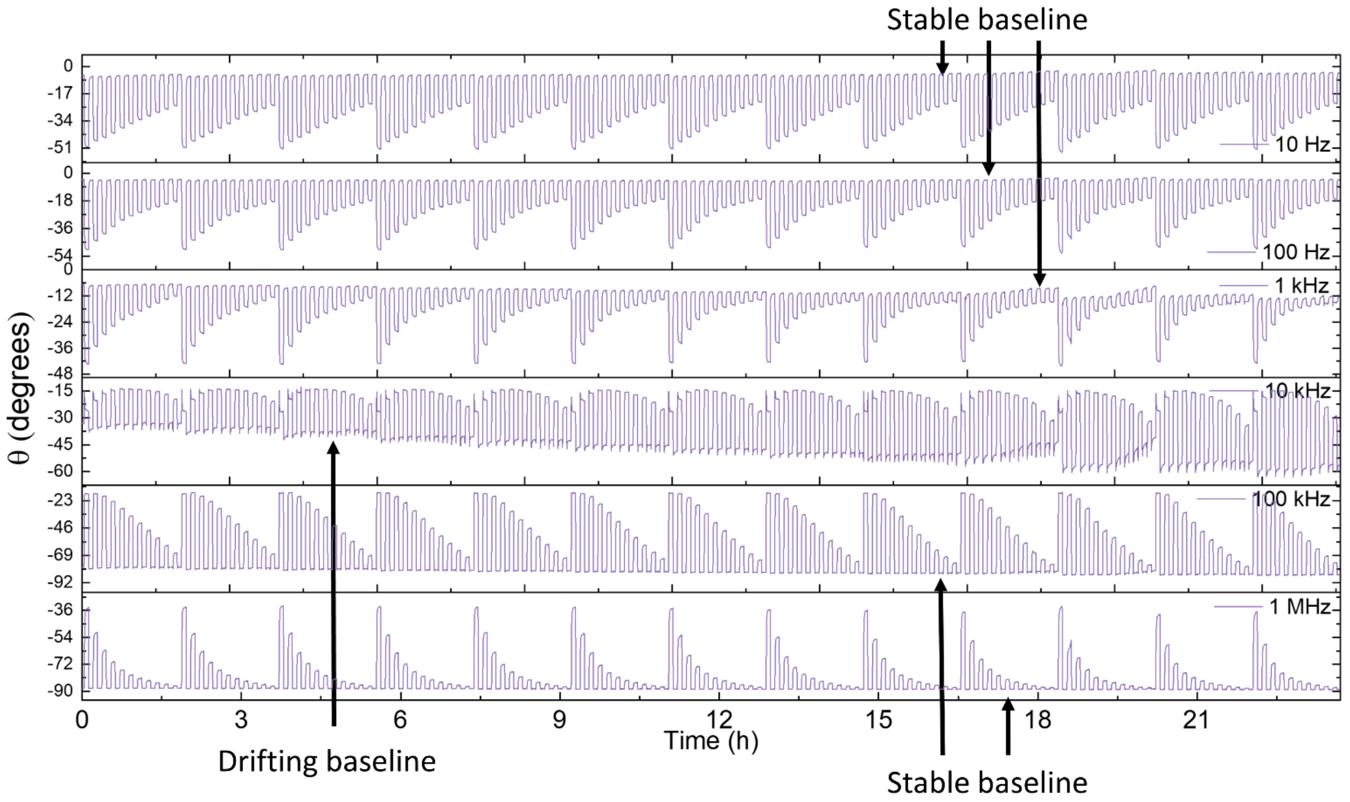

Figure S11. (a) The comparison of phase difference values of the WS_2-based sensor at various frequencies and corresponding humidity variation is given in Figure S7 (b). A WS_2-based and DHT22 sensors were operated in parallel to observe humidity variation in the chamber. The 13 cycles of varying humidity levels (0-80% RH) passed through the sensing chamber to monitor baseline drift.

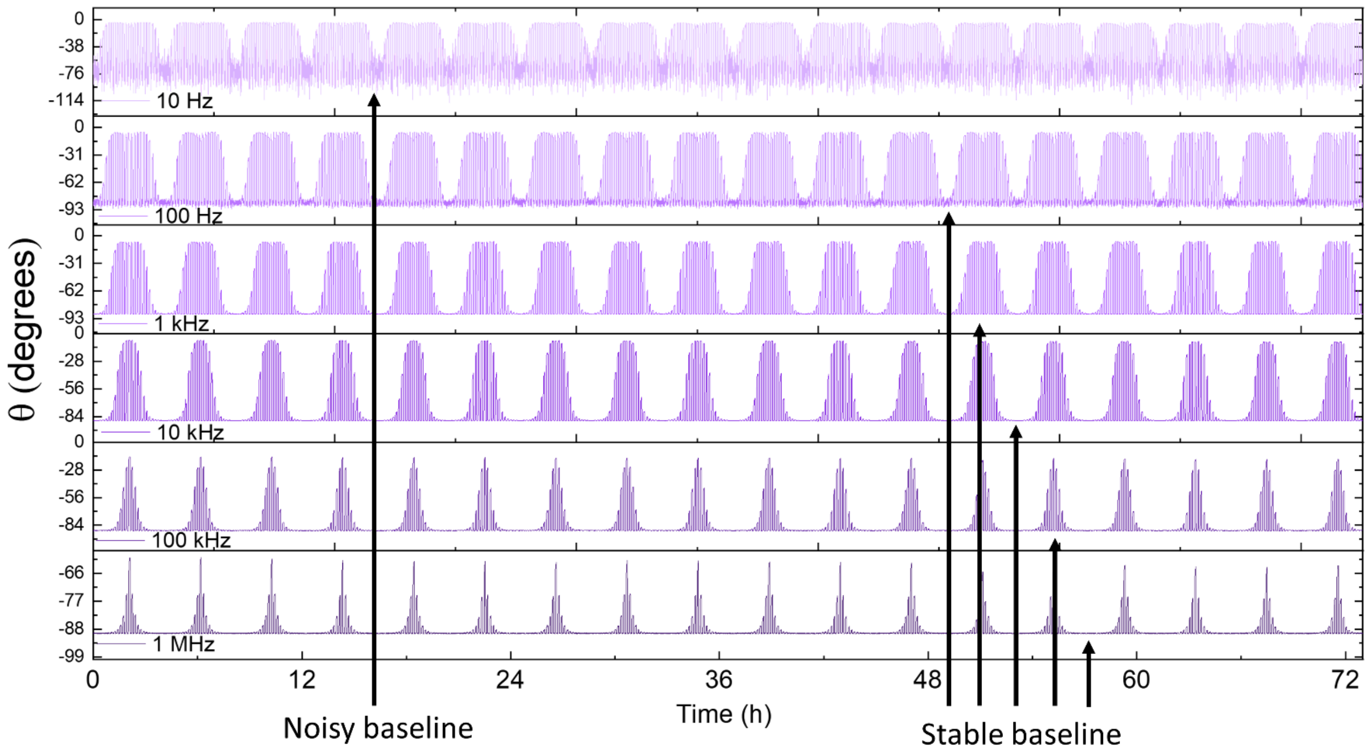

Figure S12. (a) A comparison of phase difference values of MoS_2-based sensors at various frequencies; the corresponding humidity variation is given in Figure S9 (b). MoS_2-based and DHT22 sensors were operated in parallel to observe humidity variation in the chamber. The 18 cycles of varying humidity levels (0-90% RH) passed through the sensing chamber to monitor baseline drift.

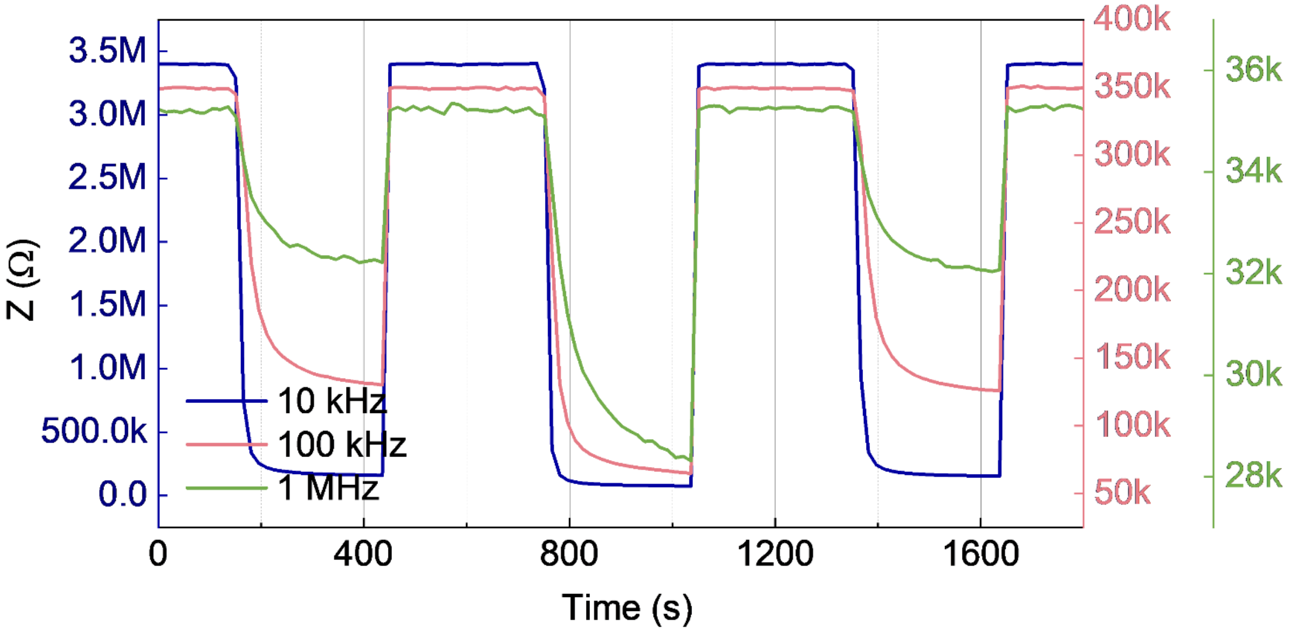

Figure S13. Comparison of response and recovery behaviour at different frequencies.

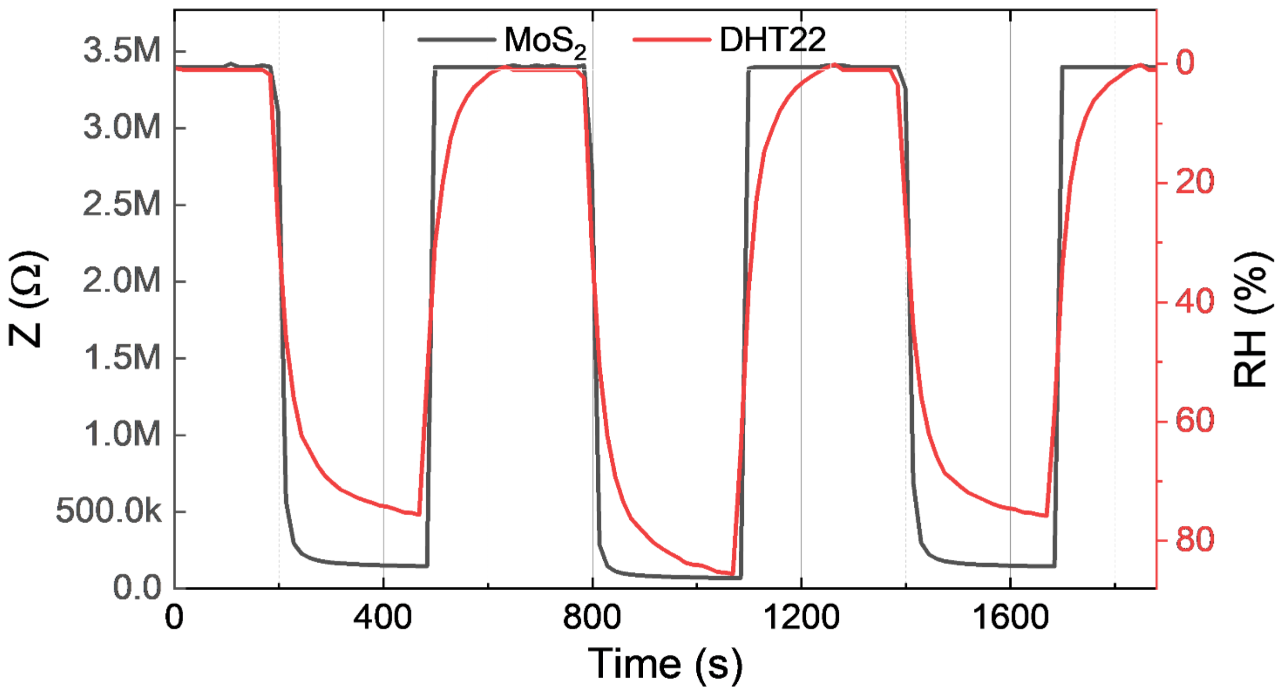

Figure S14. Comparison of response and recovery behaviour of MoS_2-based sensor compared with DHT22 sensor.

Layer (type)	Output Shape	Param #
dense_2507 (Dense)	(None, 20)	140
dense_2508 (Dense)	(None, 100)	2100
dropout_1502 (Dropout)	(None, 100)	0
dense_2509 (Dense)	(None, 500)	50500
dropout_1503 (Dropout)	(None, 500)	0
dense_2510 (Dense)	(None, 1000)	501000
dropout_1504 (Dropout)	(None, 1000)	0
dense_2511 (Dense)	(None, 500)	500500
dropout_1505 (Dropout)	(None, 500)	0
dense_2512 (Dense)	(None, 20)	10020
dense_2513 (Dense)	(None, 1)	21

Total params: 1,064,281
Trainable params: 1,064,281
Non-trainable params: 0

Figure S15. The model summary of MLP architecture

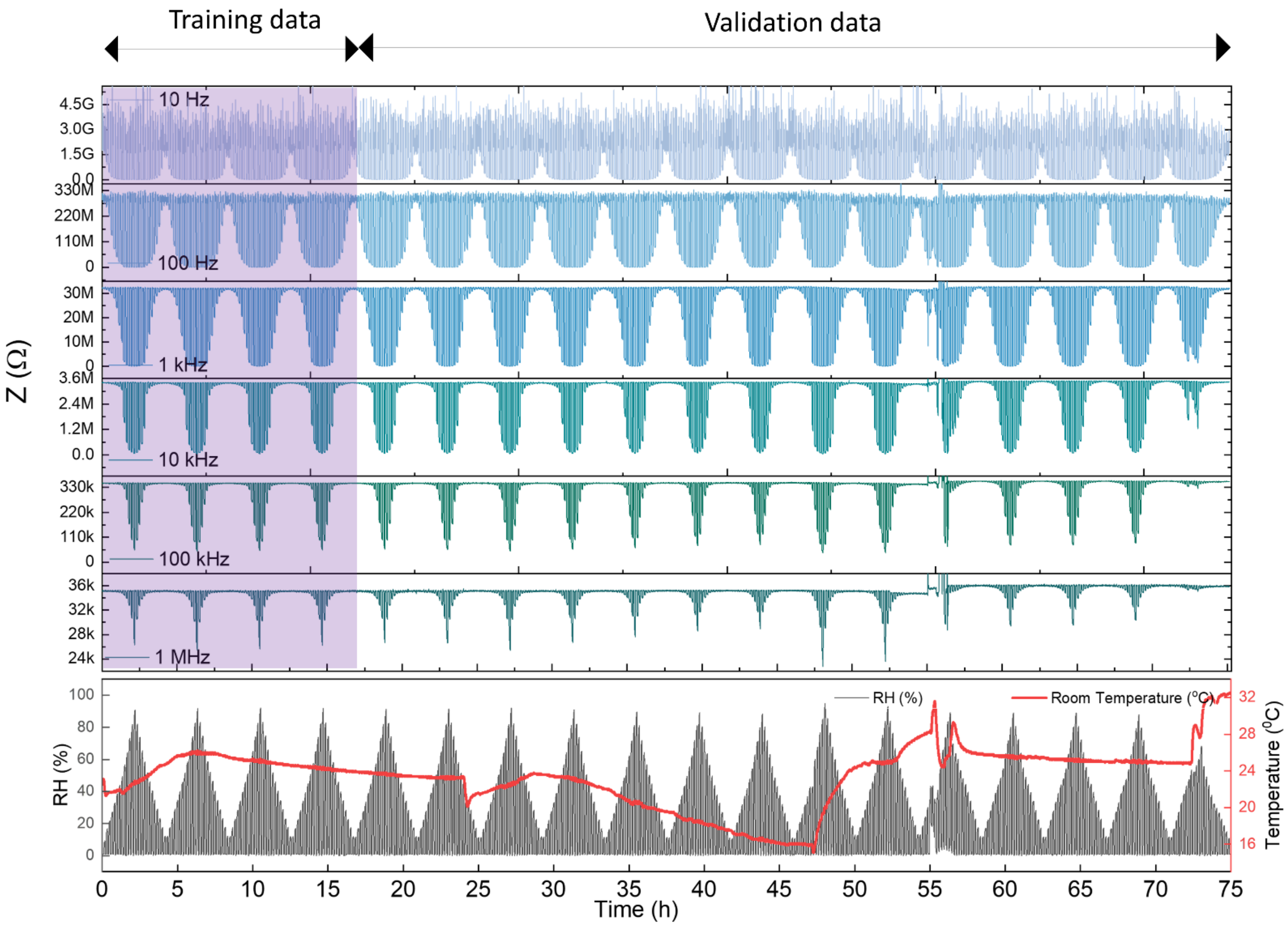

Figure S16. Data used for training and validation of MLP, 1D-CNN and LSTM

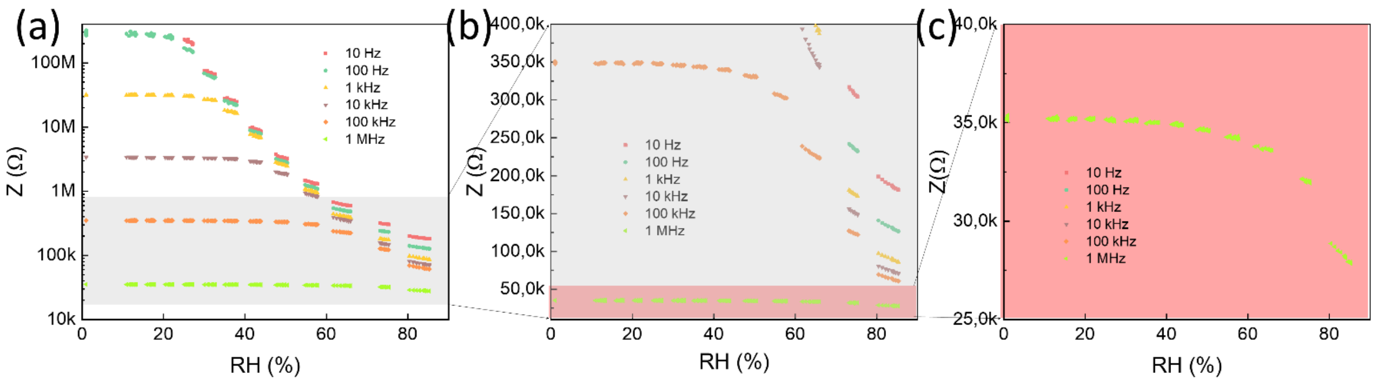

Figure S17. Comparison of impedance-based calibration curves at different frequencies.

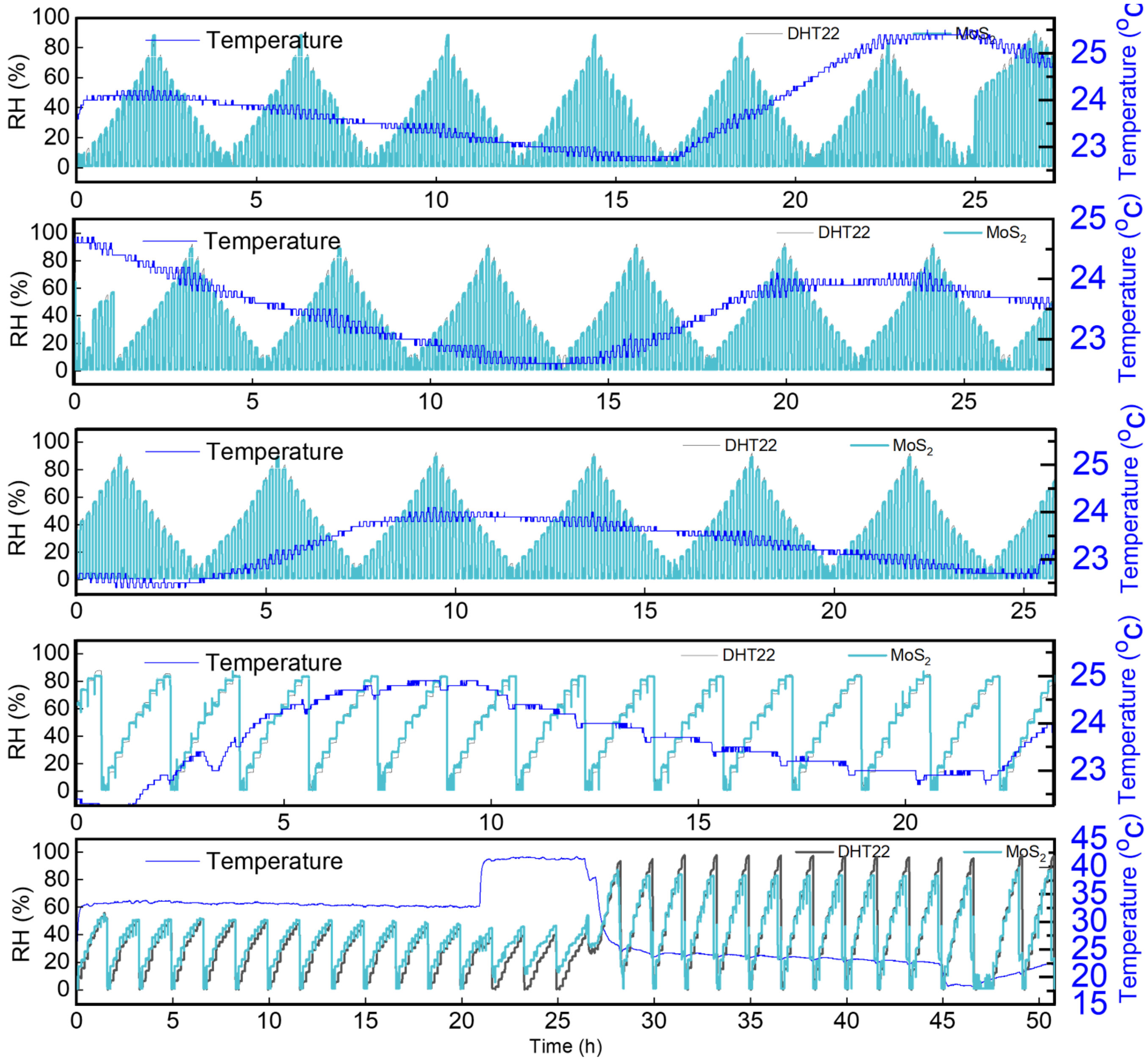

Figure S18. Reproducibility results for MoS_2-based sensor on different days.

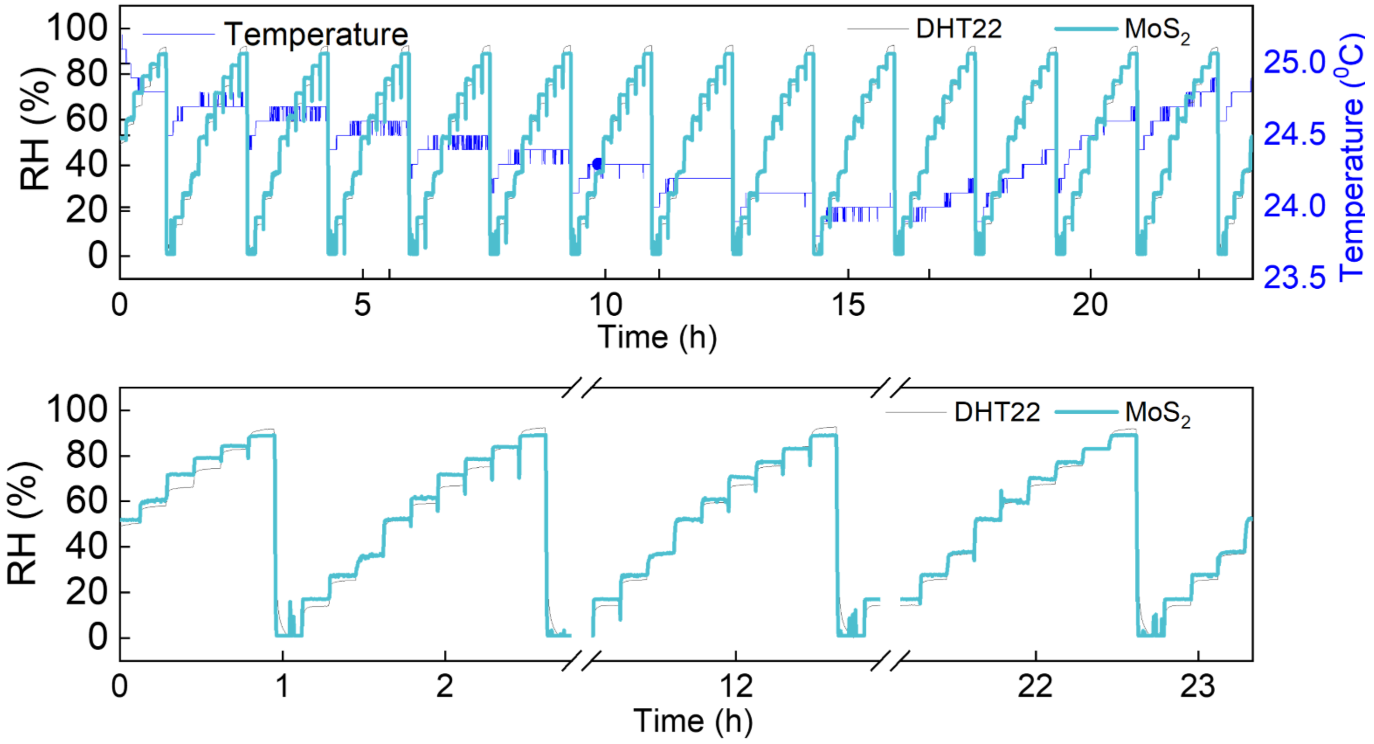

Figure S19. Reproducibility results for MoS_2-based sensor on different days.

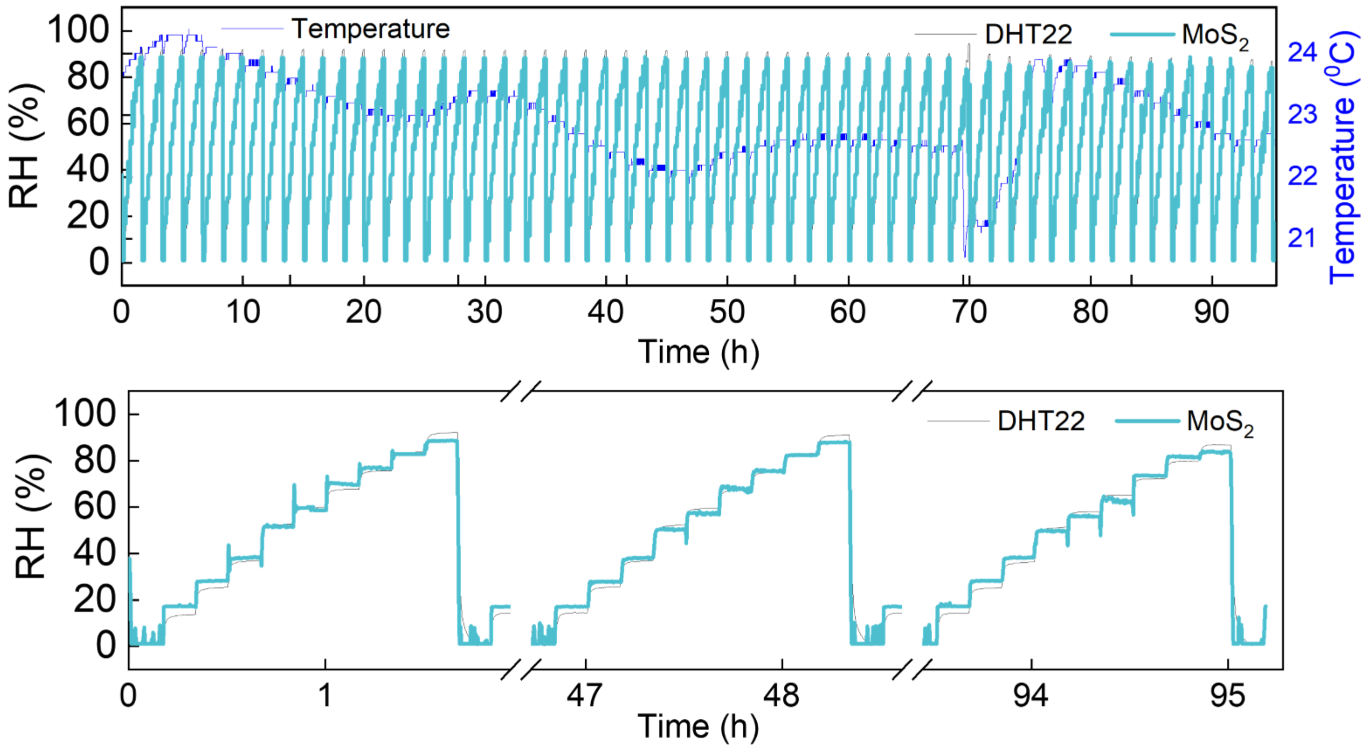

Figure S20. Reproducibility results for MoS_2-based sensors on different days.

Note 3:

In the controlled test environment, the flow rate of 1000 sccm was used and passed through a bubbler to simulate different RH concentrations. The data shown in Figure S16 was used to build the MLP model and subsequent RH estimations. The MLP model trained for 480 epochs performed well in a controlled atmosphere. The same data displayed in Figure S16 was used to train the model to estimate the ambient RH; however, the model trained for 400 epochs performed well in estimating the ambient humidity, as shown in Figure 6(c).

Model: "sequential_15"

Layer (type)	Output Shape	Param #
conv1d_45 (Conv1D)	(None, 5, 32)	96
conv1d_46 (Conv1D)	(None, 4, 64)	4,160
conv1d_47 (Conv1D)	(None, 3, 128)	16,512
flatten_15 (Flatten)	(None, 384)	0
dense_30 (Dense)	(None, 64)	24,640
dense_31 (Dense)	(None, 1)	65

Total params: 136,421 (532.90 KB)
Trainable params: 45,473 (177.63 KB)
Non-trainable params: 0 (0.00 B)
Optimizer params: 90,948 (355.27 KB)

S21. The model summary of 1D-CNN architecture. Standardization was used as pre-processing step and MAE was used as cost function during 1D-CNN training process.

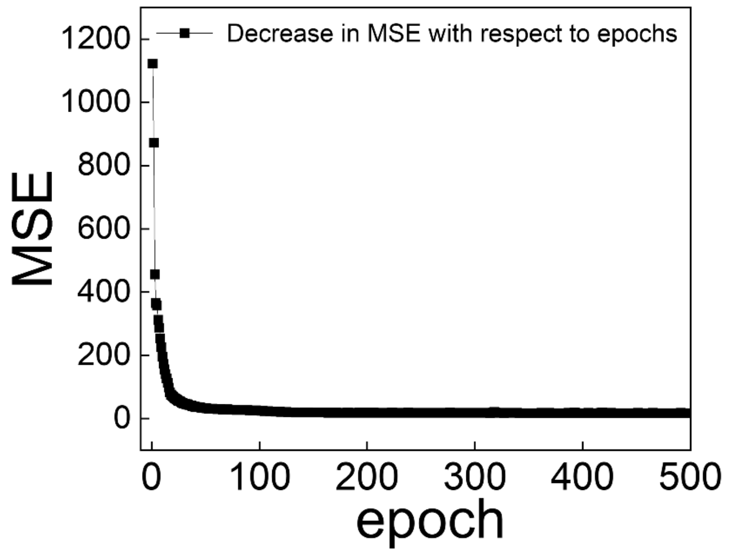

S22. A reduction in MSE with respect to epochs during the training of 1D-CNN based model

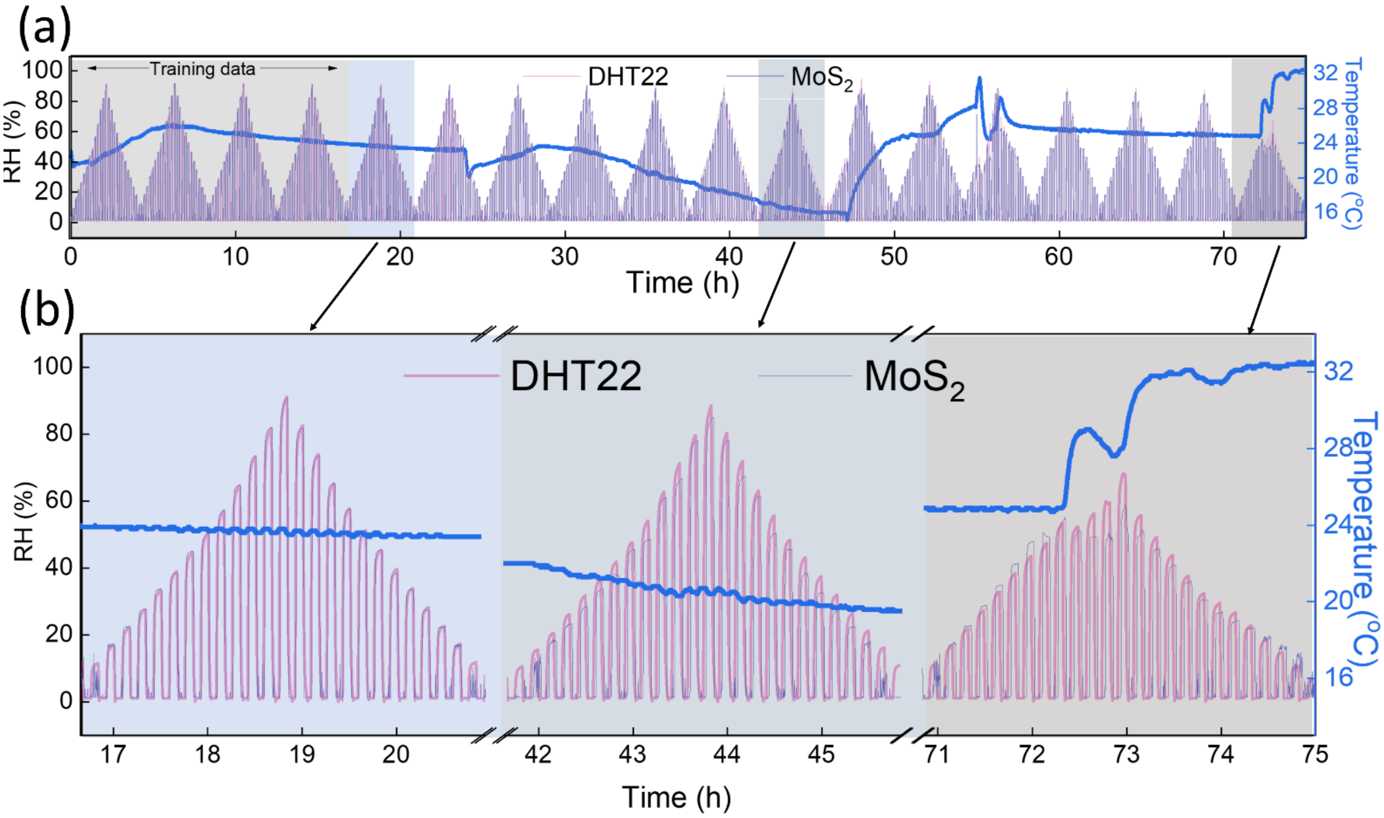

S23. (a) Predicted data for an extended duration using an 1D-CNN-based model, (b) Magnified image of estimated humidity values at different intervals. The temperature fluctuations within the sensing chamber during the measurements are also illustrated in Figures (a) and (b).

```
Model: "sequential_5"
┏━━━━━━━━━━━━━━━━━━━━━━━━━━━━━━━━━━━━━━┳━━━━━━━━━━━━━━━━━━━━━━━━━━━┳━━━━━━━━━━━━━━━━━┓
┃ Layer (type)                         ┃ Output Shape              ┃         Param # ┃
┡━━━━━━━━━━━━━━━━━━━━━━━━━━━━━━━━━━━━━━╇━━━━━━━━━━━━━━━━━━━━━━━━━━━╇━━━━━━━━━━━━━━━━━┩
│ lstm_15 (LSTM)                       │ (None, 6, 64)             │          16,896 │
├──────────────────────────────────────┼───────────────────────────┼─────────────────┤
│ lstm_16 (LSTM)                       │ (None, 6, 32)             │          12,416 │
├──────────────────────────────────────┼───────────────────────────┼─────────────────┤
│ lstm_17 (LSTM)                       │ (None, 16)                │           3,136 │
├──────────────────────────────────────┼───────────────────────────┼─────────────────┤
│ flatten_5 (Flatten)                  │ (None, 16)                │               0 │
├──────────────────────────────────────┼───────────────────────────┼─────────────────┤
│ dense_5 (Dense)                      │ (None, 1)                 │              17 │
└──────────────────────────────────────┴───────────────────────────┴─────────────────┘
 Total params: 97,397 (380.46 KB)
 Trainable params: 32,465 (126.82 KB)
 Non-trainable params: 0 (0.00 B)
 Optimizer params: 64,932 (253.64 KB)
```

S24. The model summary of LSTM architecture. Standardization was used as pre-processing step and MAE was used as cost function during LSTM training process.

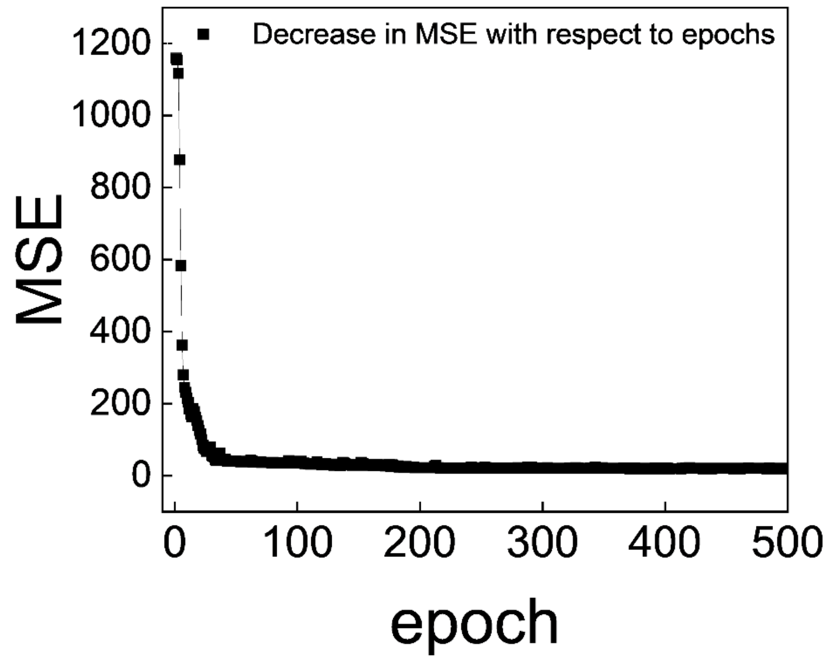

S25. A reduction in MSE with respect to epochs during the training of LSTM based model

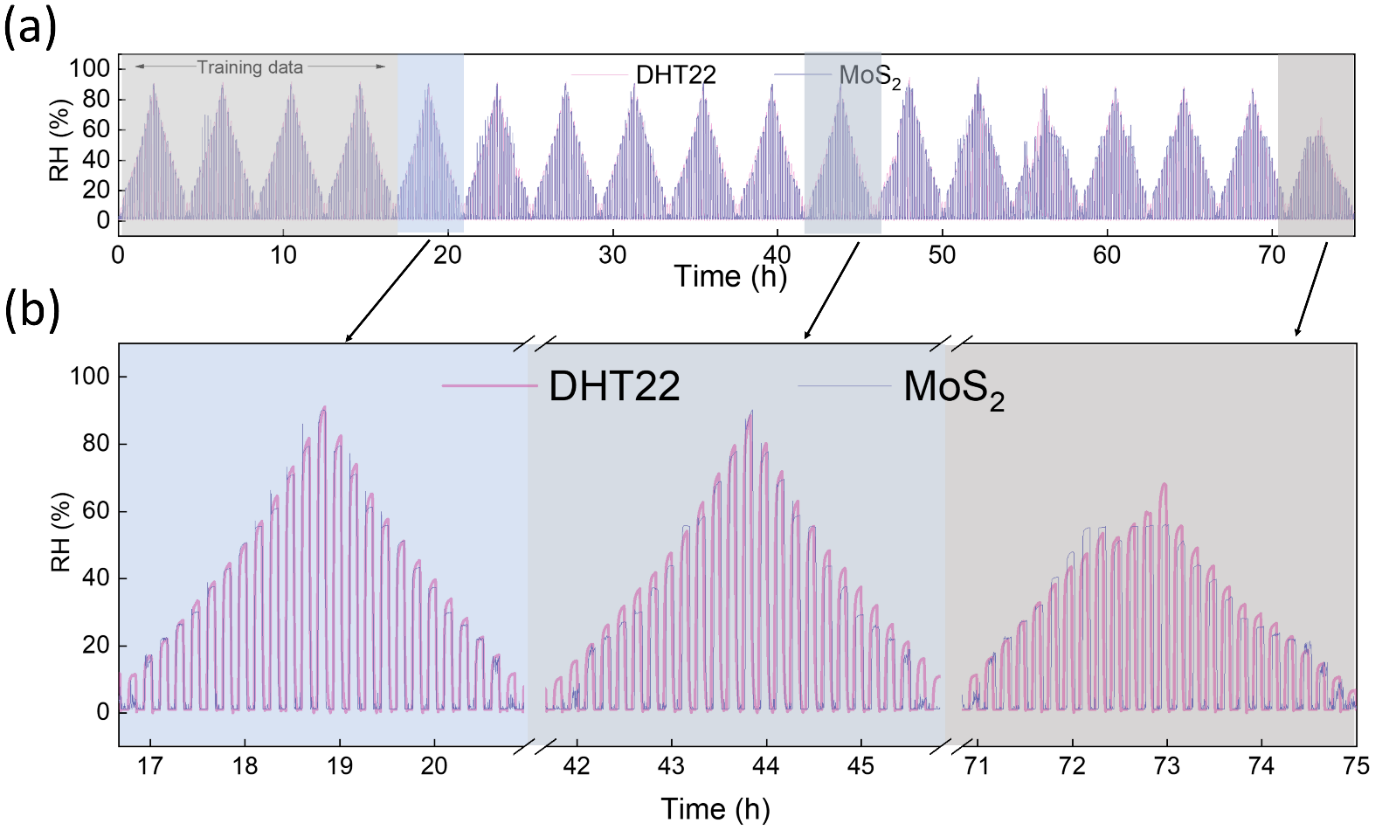

S26. (a) Predicted data for an extended duration using an 1D-CNN-based model, (b) Magnified image of estimated humidity values at different intervals. The temperature fluctuations within the sensing chamber during the measurements are also illustrated in Figures (a) and (b).

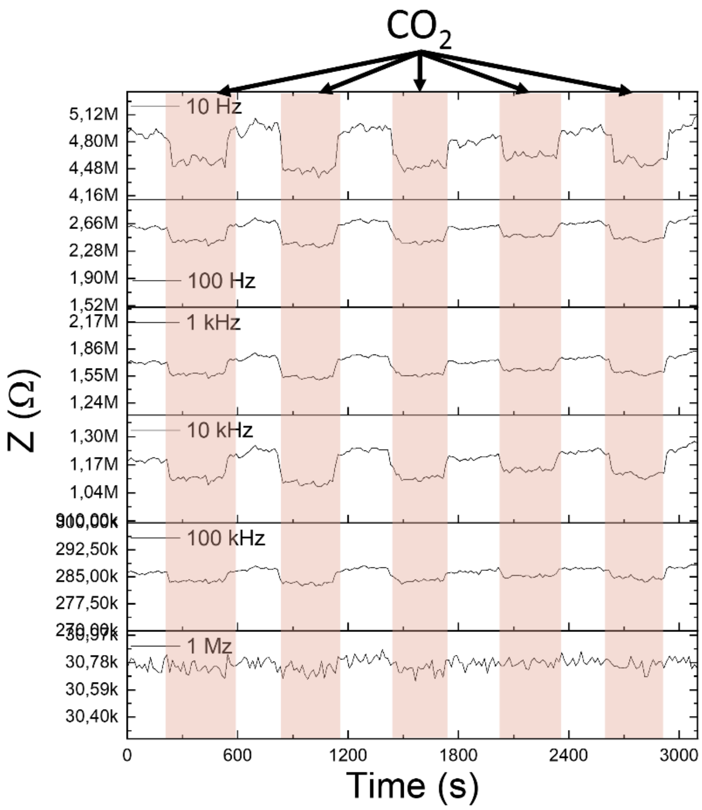

Figure S27. Impedance variation in CO_2 mixed humid atmosphere
